# Effects of geometry on large-scale tube-shear exfoliation of graphite to multilayer graphene and nanographite in water

**DOI:** 10.1038/s41598-019-45133-y

**Published:** 2019-06-20

**Authors:** Nicklas Blomquist, Majid Alimadadi, Magnus Hummelgård, Christina Dahlström, Martin Olsen, Håkan Olin

**Affiliations:** 1 Mid Sweden University, Department of Natural Sciences, Sundsvall, SE-851 70 Sweden; 2 Mid Sweden University, Department of Chemical Engineering, Sundsvall, SE-851 70 Sweden

**Keywords:** Synthesis and processing, Synthesis of graphene, Synthesis of graphene

## Abstract

Industrially scalable methods for the production of graphene and other nanographites are needed to achieve cost-efficient commercial products. At present, there are several available routes for the production of these materials but few allow large-scale manufacturing and environmentally friendly low-cost solvents are rarely used. We have previously demonstrated a scalable and low-cost industrial route to produce nanographites by tube-shearing in water suspensions. However, for a deeper understanding of the exfoliation mechanism, how and where the actual exfoliation occurs must be known. This study investigates the effect of shear zone geometry, straight and helical coil tubes, on this system based on both numerical simulation and experimental data. The results show that the helical coil tube achieves a more efficient exfoliation with smaller and thinner flakes than the straight version. Furthermore, only the local wall shear stress in the turbulent flow is sufficient for exfoliation since the laminar flow contribution is well below the needed range, indicating that exfoliation occurs at the tube walls. This explains the exfoliation mechanism of water-based tube-shear exfoliation, which is needed to achieve scaling to industrial levels of few-layer graphene with known and consequent quality.

## Introduction

To achieve cost-efficient commercial products utilizing the excellent properties of carbon nanomaterials, it is necessary to develop industrially scalable methods for the production of nanographites; such as graphene, few-layer graphene and graphite nanoplatelets^1–3^. At present, there are several available production routes of these materials, for large-scale and low-cost manufacturing, and the exfoliation of graphite seems to be the most promising method^4–6^. Using fluid dynamics to exfoliate graphite has become a popular and effective approach to achieve large quantities of nanographites and other two-dimensional materials^4,6^. High-shear mixing^2^, Vortex fluid film^7^, Jet cavitation^8–10^, Jet milling^11^, Microfluidization^12,13^ and hydrodynamic tube-shear^3,14^ are all examples of exfoliation techniques based on fluid dynamics to produce graphene, multilayer graphene and graphite nanoplatelets. Comprehensive studies of exfoliation solvents have shown that organic solvents are preferred for high yield exfoliation, due to their ability to decrease the energy barrier in the interlayer of graphite, thus requiring less force in the exfoliation process^6,11,15,16^. Hernandez *et al*.^15,16^ points out that the best candidates are organic solvents with a surface tension of approximately 40 mJm^−2^, such as N-methyl-2-Pyrrolidone (NMP) and N,N-dimethylformamide (DMF). Other organic solvents, such as isopropanol and chloroform have also shown good results, as well as some ionic liquids^6,16^. However, to promote low-cost, sustainable and environmentally friendly large-scale production, the use of expensive and in some cases toxic solvents is inappropriate and water-based solvents are preferred^3,6,13,17^. To achieve pure mechanical exfoliation, without significantly decreasing the interlayer bond strength in graphite, the process must overcome the interlayer shear strength of crystalline graphite. Ze Liu *et al*.^18^ reported a novel experimental method to directly measure the interlayer shear strength for single crystal graphite to 0.14 GPa, which is considered a benchmark for the shear strength of defect free single crystal graphite. Other reports^19,20^ show values of two to three orders of magnitude lower (0.25–2.5 MPa) and the drastic difference is suggested to be due to the presence of stacking faults between the layers. It is a challenge to precisely measure and characterize lateral size and thickness distribution of graphene from large-scale processes, since the large amount of flakes requires a substantial dataset to be considered statistically sound. Common techniques used is Raman spectroscopy, scanning electron microscopy (SEM), transmission electron microscopy (TEM), Atomic force microscopy (AFM), and optical contrast^2,3,5,21,22^. Lateral size distribution is rather simple compared to thickness since the size range is one to a few orders of magnitude larger. AFM is a precise tool to measure thickness but is time-consuming and it can be difficult to obtain precise flake thickness measurements due to wrinkled and folded flakes or an underlying solvent film that causes an instrumental offset^2,22,23^. Raman spectroscopy has become a standard tool to characterize nanographites and other 2D materials^21–24^. By analyzing the Raman spectra of graphene, it is possible to identify the layer number of flakes with few layers, up to 5 or so, for thicker flakes the Raman spectra becomes more similar to the spectra of bulk graphite^23,24^. Xiao-Li Li *et al*.^23^ demonstrated a method to identify the layer number of graphene flakes on Silicon/Silicon dioxide substrates in Raman by comparing the intensity ratio of the Si peak of the Si/SiO_2_ substrate underneath the graphene flakes, and the Si peak from the bare Si/SiO_2_ substrate. This method claims to be robust and fast and seems to be limited only by the laser spot size, which must be smaller than the area of the measured flake. In previous work^3^ we have demonstrated a scalable and low-cost industrial route to produce nanographites based on hydrodynamic tube-shearing in water suspension. The initial production rate was as high as 500 gh^−1^ and the system design allows easy scaling by adding parallel shear zones together with increased flow rate. The shear zone, a straight tube, resulted in a wide flake-size distribution of wrinkled or partly wrinkled nanometer-thick flakes. These properties have been shown to have great potential in energy storage applications where high electrical conductivity is needed together with high surface area^25–27^. However, for a deeper understanding of the exfoliation mechanism in this system, how and where the actual exfoliation occurs must be elucidated. The current report is a study of the effect on shear zone geometry in hydrodynamic tube-shear exfoliation to obtain a deeper understanding of the exfoliation mechanism and the requirements needed to achieve efficient large-scale mechanical exfoliation to nanographites in water suspensions.

## Results and Discussion

### Fluid simulation

**Figure 1:**
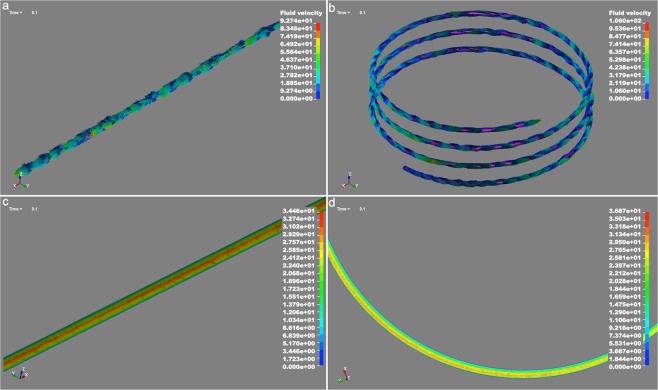
Fluid velocity in S1 and S2 for pass 0. (**a**) The fluid velocity vectors in S1. (**b**) The fluid velocity vectors in S2. (**c**) The contours of the average velocity at the central region of the length of S1. (**d**) The contours of the average velocity of S2 at a region far from the tube entrance.

Two geometries were investigated: straight tube geometry called S1 and helical coil tube called S2, both with the same length and diameter. The change in the average pressure at the tubes entrance implies that a steady state is gradually established after approximately *t* = 0.02 s in both geometries. On average, the fluid velocity was 6.5% higher in S2 compared to S1. The flow at the entrance of the tubes was calculated to be 3.85 Lmin^−1^ which is lower than the measured experimental flow, that is 4.95 Lmin^−1^. This implies that the constant fluid velocity of 26.3 ms^−1^ may underestimate the flow behavior. Figure 1a,b show the fluid velocity vectors in S1 and S2, respectively.

**Figure 2:**
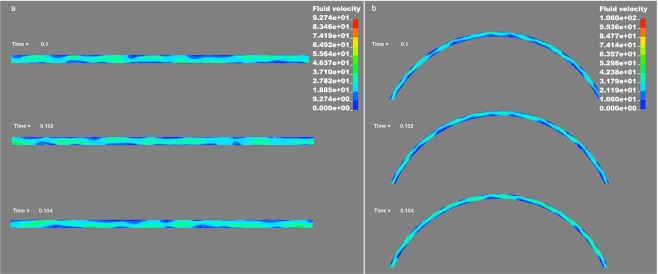
Magnitude of instantaneous fluid velocity at *t* = 0.100 s, *t* = 0.102 s and *t* = 0.104 in (**a**) S1 and (**b**) S2.

The Reynolds numbers were calculated using the maximum average velocity and were 1.6 × 10^5^ for S1 and 1.7 × 10^5^ for S2. The Reynolds number values suggest that the flows in both geometries were turbulent, which was also understood from the flow patterns. A distinct difference between S1 and S2 is the swirling flow in S2 that continues throughout the coil. Figure 1c shows the contours of the average velocity at the central region of the length of S1 at *t* = 0.1 s and Fig. 1d shows the contours of the average velocity at *t* = 0.1 of S2, at a region far from the tube entrance. Comparing these two figures reveals details of the flow patterns in the two geometries: on average, the velocity profile gradient in the straight tube is parabolic with a maximum at the centerline of the tube, while the velocity profile in the helical coil tube has a maximum close to the tube wall at the side of the outer helix radius. Figure 2a,b show the magnitude of instantaneous fluid velocity in S1 and S2 respectively, at *t* = 0.100 s, *t* = 0.102 s, and *t* = 0.104 s. The mixing effect is clearly seen in the irregular velocity fluctuations. The expansion and reduction of the regions where the fluid velocity is close to zero is notable.

The measured flow at the entrance of the tubes were used together with equation 1 to calculate the laminar shear rate and shear stress. The average fluid velocity from Fig. 1c,d and the local fluid velocity at the tube wall from Fig. 2a,b were used together with equations (2 and 5) to calculate the average wall shear stress and local wall shear stress in the exfoliation process. The result is shown in Table 1. The corrected values (corr.) are calculated with consideration of the flow difference between the numerical simulation and experimentally measured flows. The results demonstrate that the laminar shear stress contribution is insufficient for exfoliation while the turbulent local wall shear stress is in the range to overcome the interlayer shear strength of graphite. This also indicates that the exfoliation occurs at the tube wall and not in the flow gradient. More details and figures from the simulation can be found in the supplementary information.

**Table 1:** Shows the laminar and turbulent shear stress contribution for both geometries in the tube-shear exfoliation system. The corrected values (corr.) are calculated with consideration of the flow difference between the numerical simulation and experimentally measured flows. The interlayer shear strength for graphite is in the range between 0.25 MPa to 0.14 GPa, where the upper range is for defect free single crystal graphite.

Laminar and turbulent shear stress contribution		
	S1	S2
Shear rate (laminar)	1.1 × 10^5^ *s*^−1^	3.2 × 10^5^ *s*^−1^
Shear stress (laminar)	4.6 × 10^1^ Pa	1.4 × 10^2^ Pa
Average wall shear stress (turbulent)	6.8 × 10^4^ Pa	8.0 × 10^4^ Pa
Local wall shear stress (turbulent)	2.7 × 10^6^ Pa	3.5 × 10^6^ Pa
Average wall shear stress, corr. (turbulent)	1.1 × 10^5^ Pa	1.3 × 10^5^ Pa
Local wall shear stress, corr. (turbulent)	4.4 × 10^6^ Pa	5.7 × 10^6^ Pa

### Exfoliation

During exfoliation both suspensions was forced through their corresponding shear zone 10 times (10 passes) followed by immediate dilution and spin coating to prepare the samples for characterization.

#### Flake size and thickness distribution

**Figure 3:**
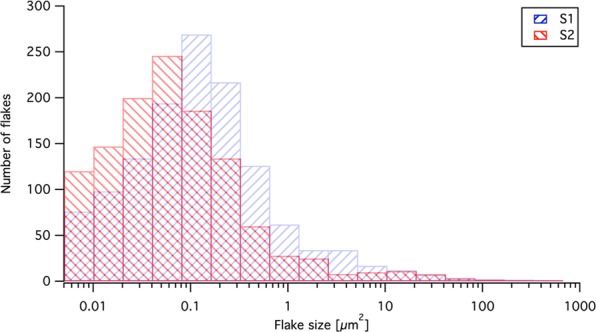
Histogram of the flake size distribution after 10 passes for S1 and S2 from SEM image analysis. The flake size distribution is presented in flake area and the x-axis is logarithmic and the well-width is doubled for each well starting at 0.005 *μ*m^2^.

Figure 3 shows the flake size (lateral) distribution for S1 and S2 after 10 passes from SEM image analysis. The flake size distribution has a similar shape for both geometries, but the distribution for S2 shifts to a smaller flake size compared to S1. The flake size distribution peaks at a flake area of approximately 0.12 *μ*m^2^ for S1 and approximately 0.06 *μ*m^2^ for S2. This could be explained by the more efficient geometry of S2 compared to the straight tube S1. The S2 tube is designed to create more turbulent flow, and closer to the tube wall, to be able to exceed the shear stress levels required for proper exfoliation.

**Figure 4:**
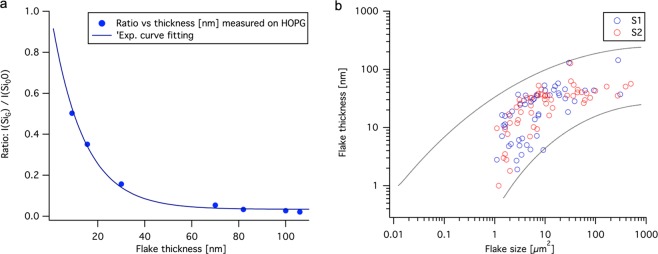
(**a**) The ratio I(SiG)/I(Si0) as a function of the flake thickness. The blue (°) markers shows experimental data from the tape exfoliated HOPG flakes in the Horiba XploRA PLUS Raman system as a function of the measured thickness in nm together with a fitted trend line. (**b**) Flake thickness distribution for S1 and S2 after 10 passes together with one upper and one lower trend line.

To determine the flake thickness distribution we followed the recent reported method by Xiao-Li Li *et al*.^23^. To calibrate the system, we measured the Raman intensity ratio I(SiG)/I(Si0) of tape exfoliated highly oriented pyrolytic graphite (HOPG) flakes with known thickness from AFM. Figure 4a shows the ratio I(SiG)/I(Si0) as a function of the flake thickness for the tape exfoliated HOPG flakes, together with a fitted trend line. When plotting the HOPG flake Raman intensity ratio as a function of the thickness, we obtain an almost identical trend line as Xiao-Li Li *et al*. but with an offset. The offset is approximately a factor 3, which fits well with the graphene layer spacing of 0.333 nm. This offset can be due to the use of a different Raman spectroscopy system compared to Xiao-Li Li *et al*. and that no instrumental offset has been subtracted in the AFM measurements in this experiment, which gives a higher thickness value. The trend line from the HOPG flake Raman intensity ratio was used to determine the flake thickness distribution of the tube exfoliated flakes from both shear zones, as plotted in Fig. 4b. The blue plot shows experimental data from the tape exfoliated HOPG flakes in a Horiba XploRA PLUS Raman system, with a 523 nm and 100 mW green laser, as a function of the measured thickness in nm together with a fitted trend line. All of the Raman spectra’s are included in the supplementary information document.

The thickness distributions are similar for both geometries. The trend shows that the flake thickness is found to be thinner with smaller flake size (that is, the flake surface area) but simultaneously the variation of the thicknesses distribution broadens, see Fig. 4b. The results demonstrates that flakes with sizes smaller than a few square micrometers have a thickness in the sub- or few nanometer range thus indicating that the process is efficient enough to produce multilayer graphene. The shear stress from the laminar flow will not be sufficient to create high-enough shear stress for exfoliation. In the simulation of the geometries, the turbulent wall shear stress achieved is above the threshold for exfoliation, but still in the lower end of the range. This means it is possible that graphite flakes with higher crystallinity still might not be exfoliated in the process, which could explain the measured broadening in the flake thickness distribution. If a simple exfoliation model is assumed to describe the process, wherein the flakes are continuously split in half, the outcome would be a flake thickness distribution that grows narrower over time as the exfoliation process continues. However, the results demonstrates that there is a broadening in the thickness variation when the flakes become smaller, indicating that a simple exfoliation model is not sufficient to describe the results. The reason for this broadening of thickness variation have not been investigated in detail in this study, but based on the previous discussion it is suggested that it must be either a more complex exfoliation process or several competing processes. A subgroup of competing processes could be that the initial expanded graphite material may have contained size dependent fractures or crystal faults, and not a simple distribution of similar particles. This would lead to particles affected differently by the process and thus result in a broadening of the flake thickness distribution.

## Conclusion

This study investigates the effects of geometry on tube-shear exfoliation of multilayer graphene and nanographite in water. Two geometries were investigated: straight tube and helical coil tube, both with the same length and diameter. The simulation shows that turbulent flow is present in both geometries and that induced shear stress is larger in the helical coil tube compared to the straight version. This is also visible in the experimental results wherein the flake size distribution is shifted to smaller flakes for the helical coil tube. Moreover, the simulation shows that the laminar shear stress contribution is insufficient for exfoliation but that the turbulent local wall shear stress is in the required range, thus indicating that the exfoliation most likely occurs at the tube wall and not in the flow gradient. The difference in geometry does not indicate any significant differences in flake thickness distribution but since the helical coil tube achieves a larger number of smaller flakes, the amount of multilayer graphene produced is higher for the helical coil tube and it is therefore more efficient.

## Methods

### Fluid simulation

**Figure 5:**
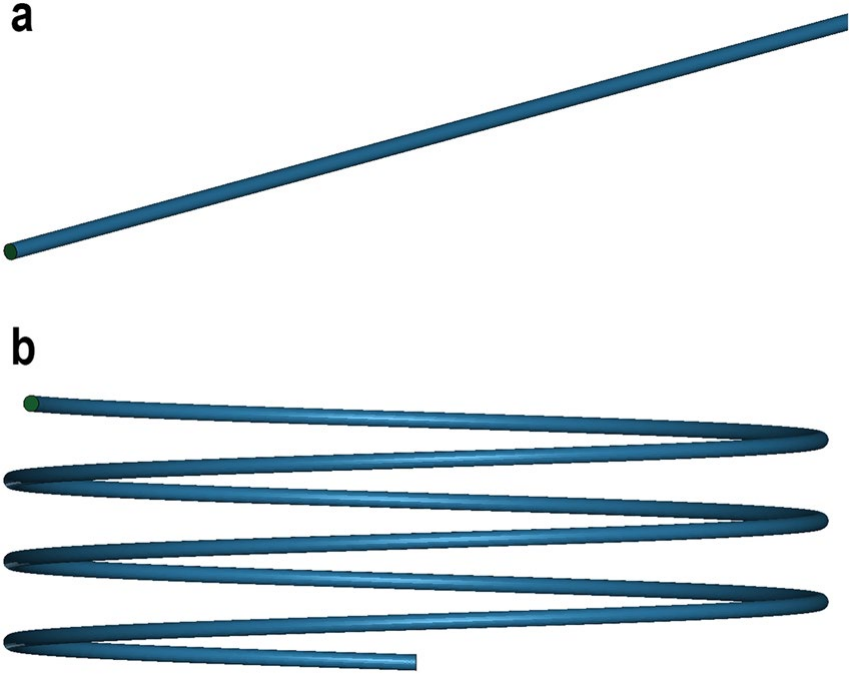
3D sketches of the tubes shear zones where (**a**) is the straight S1-version and (**b**) is the helical coil tube called S2. Both tubes had a diameter of 2 mm and length 1000 mm. The helical coil tube had a helix diameter of 100 mm with a pitch of 10 mm.

Due to the different centrifugal forces across the width of a curved tube, caused by fluid elements moving with different axial velocities, the flow becomes more complex compared to a straight tube and secondary flows are introduced^28–30^. We simulated such flow behavior in a helical coil tube and a straight tube and compared the results to experimental data. The simulation is based on the fluid, with experimentally measured viscosity, without regard to particle interaction. For the numerical simulation, the explicit time integration finite element code LS-DYNA (Livermore Software Technology Corporation, USA) was used. The straight tube was modeled by a three-dimensional (3D) tube 2 mm in diameter and 1000 mm in length. The helical coil tube had a 2 mm tube diameter, a 100 mm helix diameter, and a 10 mm helix pitch. The selection of the mesh size was based on a compromise between the simulation accuracy and the computation efficiency. Ideally, finer mesh was desired, however, even with the utilized mesh size, the total number of elements was over 1.6 × 10^6^. This is because the length and diameter of the simulation domain in each case were the real size. The mesh density for S1 and S2 were 555 and 535 tetrahedral elements per mm^3^, respectively. In Fig. 5, 3D sketches of the tubes are displayed. The flow rate and the fluid viscosity were adopted from the experiments. A constant velocity of 26.3 ms^−1^ was applied at one end of the tubes for all of the simulations. The pressure at the other end of the tubes was atmospheric. The non-slip boundary condition was applied to the tube walls. The fluid viscosity was adopted to be 0.44 mPas, which is the viscosity of the 2% graphite suspension before exfoliation (pass 0). The viscosity of the suspension were measured using an Anton Paar Physica MCR 300 Rheometer. The fluid density was assumed to be 1000 kgm^−3^. The simulation time was set to 0.2 s.

#### Shear stress calculations

To overcome the interlayer shear strength of the graphite, the induced shear stress in the exfoliation process must exceed 0.14 GPa^18^ for defect-free single crystal graphite and at least 0.25 MPa^19,20^ for graphite containing stacking faults. The general wall shear stress,*τ*_*w*_, induced in the shear zone geometry by the fluid is provided by equation (1).

1 $$\tau (w)={\mu \frac{\partial U}{\partial y}|}_{y=0},$$

where *μ* is the dynamic viscosity of the flow, U is the flow velocity along the boundary and y is the height above the boundary. For turbulent flow in a rough pipe, the mean shear stress is provided by equation (2)^31^, were *f* is the Darcy friction factor. The Darcy friction factor can be solved iteratively using the Colebrook equation or directly calculated (within a few percent) by equation (3)^32^.

2 $${\tau }_{w.avg.}=f\frac{\rho {V}_{avg}^{2}}{8},$$

3 $$\frac{1}{\sqrt{f}}=-1.8\,\mathrm{log}[\frac{6.9}{Re}+{(\frac{\varepsilon /D}{3.7})}^{1.11}],$$

where *ρ* is the fluid density, *V*_*avg*_ is the mean fluid velocity, *ε* is the tube wall surface roughness (2 × 10^−5^ m), *D* is the tube diameter and *Re* is the Reynolds number which is provided by equation (4)^31^

4 $$Re=\frac{\rho {V}_{avg}^{2}D}{\mu },$$

where *μ* is the dynamic fluid viscosity and D is the tube diameter. The local wall shear stress, *τ*_*w*_, in the turbulent flow can be calculated using the local fluid shear velocity, u_*_, at the tube wall by equation (5)^31^.

5 $${\tau }_{w}=\rho {u}_{\ast }^{2}$$

### Exfoliation

To prepare the suspension for exfoliation, 20 gL^−1^ thermally expanded graphite (EXG 9840, Graphit Kropfmühl, Germany) was mixed in water with an addition of 2 wt% polyacrylic acid (SIGMA-ALDRICH, USA), in relation to the graphite amount, as dispersant. Two equal suspensions were prepared, each with a volume of 20 L. The pH was measured to 2.5 in both suspensions. The expanded graphite has initially low pH when mixed with water, due to residual acid from the manufacturing process. To exfoliate, the suspension was forced by a high-pressure pump through a 1 m long straight tube with an inner diameter of 2 mm. The flow rate was held constant at 4.95 Lmin^−1^. Similar suspension was forced through a 1 m long helical coil tube with an inner tube diameter of 2 mm, a helix diameter of 100 mm, and a helix pitch of 10 mm. The flow rate was held constant at 4.95 Lmin^−1^ in both geometries. This procedure was repeated 10 times (10 passes) for each case, for a total production rate of 594 gh^−1^. Samples of the suspensions were obtained before exfoliation (pass 0) and directly after the tube at the tenth pass (pass 10) from both geometries to examine the change in the flake structure. The samples were directly diluted to 0.015% with ethanol to prevent agglomeration and facilitate the preparation of specimens for material characterization in SEM, AFM and Raman spectroscopy.

### Sample preparation and material characterization

To analyze the flake thickness and size distribution in the suspensions, 100 *μ*l of the diluted suspensions (0.015%) was spin coated onto silicon wafers at a speed of 500 rpm for 10 s followed by 3000 rpm for 20 s. For flake size distribution in SEM 1 cm^2^ highly doped silicon wafer chips from Ted Pella (16006) was used, mounted on an aluminum SEM sample stub. For thickness measurement and graphene layer determination 4 cm^2^ silicon wafer chips with 90 nm silicon dioxide layer (Graphene Supermarket, USA) was used to obtain better optical contrast. Six wafer chips of both sizes was prepared for each suspension.

#### Flake size distribution

Flake size distribution was investigated using a field emission scanning electron microscope (TESCAN MAIA3-2016) at 3 kV. The 1 cm^2^ silicon wafer were divided into 9 (3 × 3 mm) sections (3 rows and 3 columns). To obtain a fair representation of the flakes the center and left center section was imaged. Each section was analyzed with 900 (30 × 30) images using a view field of 100 *μ*m (magnification: 5537x). The SEM instrumental resolution was 1 nm and the exfoliated material had a flake length and width in the range from approximately 100 nm to 10 *μ*m. The SEM viewfield and image resolution was set to generate a pixel size of 49 nm, which was sufficient to determine the flake size distribution by image analysis. A total amount of 2648 flakes was analyzed. To determine the flake size distribution, the flakes were characterized in size by image analysis using ImageJ software. The contrast and brightness settings during the image acquisition of all of the SEM images were held constant to simplify the grayscale thresholding. Before thresholding, the images were filtered three times using a median filter with a size of 1 pixel. Most of the flakes were simple to separate from the background due to the substantial difference in the intensity values, and the automatic thresholding plugin RenyiEntropy was best suited for our 16-bit images. Some of the flakes that were not thin and flat in the SEM images and shad both dark and white areas and therefore the grayscale thresholding was insufficient. These flakes were removed from the automated ImageJ algorithm and processed manually. For those flakes, an edge detection procedure was used instead of grayscale thresholding. The difference between these methods is further described in the supplementary. Finally, all of the flakes in the image data set were counted and their areas were measured. Flakes overlapping the image borders were discarded from the data set. The final statistical data set was then used for the size distribution histogram, as shown in Fig. 3.

#### Flake thickness distribution

The thicknesses of the flakes were measured using Raman spectroscopy and AFM. Raman spectra have characteristic peaks that provide information regarding the number of carbon layers present in each flake. For thicker flakes, from approximately 5 layers, the Raman spectra became more similar to the spectra of bulk graphite. AFM, although a precise tool, is too slow to analyze large data sets. Therefore, AFM was used only to verify and calibrate the Raman method in this study. Xiao-Li Li *et al*.^23^ demonstrated a robust and fast method to identify the layer number of graphene flakes on silicon/silicon dioxide substrates by comparing the intensity ratio of the Si peak of the Si/SiO_2_ substrate underneath the graphene flakes, I(SiG), and the Si peak from the bare Si/SiO_2_ substrate, I(Si0). This ratio were set in relation to the flake thickness, measured by AFM, and plotted in a Lin-Log diagram. I(SiG)/I(Si0) slightly differs with the Silicon oxide layer thickness, laser wavelength and the numerical aperture of the Raman microscope. Both Raman spectroscopy and AFM were used to determine this ratio in the current system, with tape exfoliated flakes of HOPG with different thicknesses. The laser wavelengths and silicon oxide layer thicknesses used were the same as Xiao-Li Li *et al*. Curve fitting was used to generate a trend line, which is plotted in Fig. 4a. The AFM measurements were performed in room temperature at atmospheric pressure with a Nanosurf Easyscan 2 AFM using the tapping mode and ACLA SPM probes (APP NANO, USA). The Raman spectra of the tube-shear exfoliated flakes were mapped using a Raman microscope (Horiba XploRA PLUS, 100 mW solid state laser with 532 nm excitation wavelength, 1000x optical magnification, and NA: 0.9) in the frequency range of 400–3000 cm^−1^. Line scans and maps were performed over the flakes with a step size of 0.1 *μ*m to 0.5 *μ*m. The spectral focus was adjusted in every point to maximize the silicon signal and correct for any substrate slope. The intensity I(Si_0_) were taken from the points outside of the flake in the scan. The mean value of 3 points were used for both I(Si_*G*_) and I(Si_0_) for each flake. The ratio was calculated, translated to thickness with the trend line in Fig. 4a and plotted in Fig. 4b.

## Supplementary information

Supplementary information - Effects of geometry on large-scale tube-shear exfoliation of graphite to multilayer graphene and nanographite in water
