# Supplementary material for: Effects of geometry on large-scale tube-shear exfoliation of graphite to multilayer graphene and nanographite in water
Source: Sci Rep. 2019 Jun 20;9:8966. doi: 10.1038/s41598-019-45133-y (PMC6586880; doi:10.1038/s41598-019-45133-y)
Supplement: Supplementary file 1 — Supplementary information - Effects of geometry on large-scale tube-shear exfoliation of graphite to multilayer graphene and nanographite in water [file 41598_2019_45133_MOESM1_ESM.pdf]

Supplementary information

Effects of geometry on large-scale tube-shear exfoliation of graphite to multilayer graphene and nanographite in water

Nicklas Blomquist[1,*], Majid Alimadadi[1], Magnus Hummelgård[1], Christina Dahlström[2], Martin Olsen[1] and Håkan Olin[1].

[1]Mid Sweden University, Department of Natural Sciences, Sundsvall, SE-851 70, Sweden
[2]Mid Sweden University, Department of Chemical Engineering, Sundsvall, SE-851 70, Sweden
*Nicklas.blomquist@miun.se

Table of content

1 Fluid simulation **2**
- 1.1 Viscosity measurements 2
- 1.2 Fluid behavior 2

2 Raman spectroscopy **5**
- 2.1 Flake thickness determination from Raman 5
- 2.2 Summary of raman spectrums 6
  - 2.2.1 Raman spectra for S1 and S2 7
  - 2.2.2 Optical images of the measured particles in raman 13

3 SEM characterization **15**
- 3.1 Image analysis 15
- 3.2 Initial material 15

References **16**

1 Fluid simulation

The explicit time integration finite element code LS-DYNA (Livermore Software Technology Corporation, USA) was used for the simulation. Two geometries were modeled: a straight tube (S1) of the diameter 2 mm and length 1000 mm and a helical coil tube (S2) with a tube diameter of 2 mm, a helix diameter of 100 mm and a helix pitch of 10 mm.

1.1 Viscosity measurements

In order to utilize in the simulation, the dynamic viscosity of 2% graphite suspension was measured on the initial material and after each pass using an Anton Paar Physica MCR 300 Rheometer. In figure 1 viscosity is plotted as a function of shear rate.

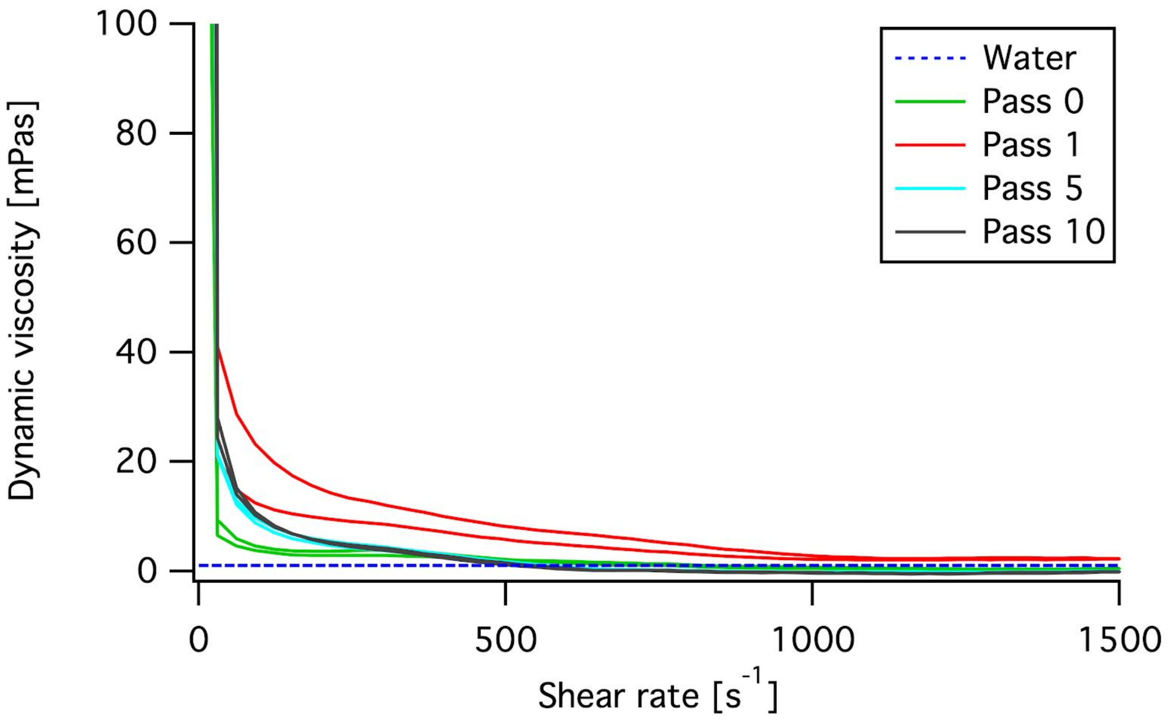

Figure 1: Dynamic viscosity as a function of shear rate for water and the graphite suspension after pass 0, 1, 5, and 10 in the straight geometry.

The shear thinning behavior of the graphite suspension is evident which is attributed to the alignment of particles. At high shear rates (>1000 s^{-1}), however, the viscosity becomes relatively constant. After the first pass of shear exfoliation, the suspension becomes thicker probably due to particles entanglement. In figure 1 this behavior is seen in pass_1 curve which is above the curves of other passes at all shear rates. For the simulation a constant fluid viscosity was adopted, i.e., 0.44 mPas which is the viscosity of the 2 % graphite suspension before exfoliation (pass_0) and 2.41 mPa.s which is the viscosity of the 2% graphite suspension after the first run (pass_1) of the exfoliation process. Both values were the average of viscosity values at >1000 s^{-1}.

1.2 Fluid behavior

Figure 2 shows the average pressure at the tubes inlet throughout the simulation time. A steady state is gradually established after about t = 0.02 s. The inlet pressure at the straight tube (S1) is higher than that of the helical coil tube (S2) and proportionally changes with the change of viscosity. The flux at the entrance of the tubes was calculated to be 3.85 l min^{-1}

which is lower than the measured experimental flux, i.e., 4.95 l min^{-1}. This implies that the constant fluid velocity 26.3 ms^{-1} may underestimate the flow behavior. The Reynolds number of the four systems were calculated to be $1.6x10^5$ and $3x10^4$ in S1 and $1.7x10^5$ and $3.3x10^4$ in S2 respectively for pass_0 and pass_1. Based on the Reynolds number and revealed by the simulation results the flows in the tubes were turbulent.

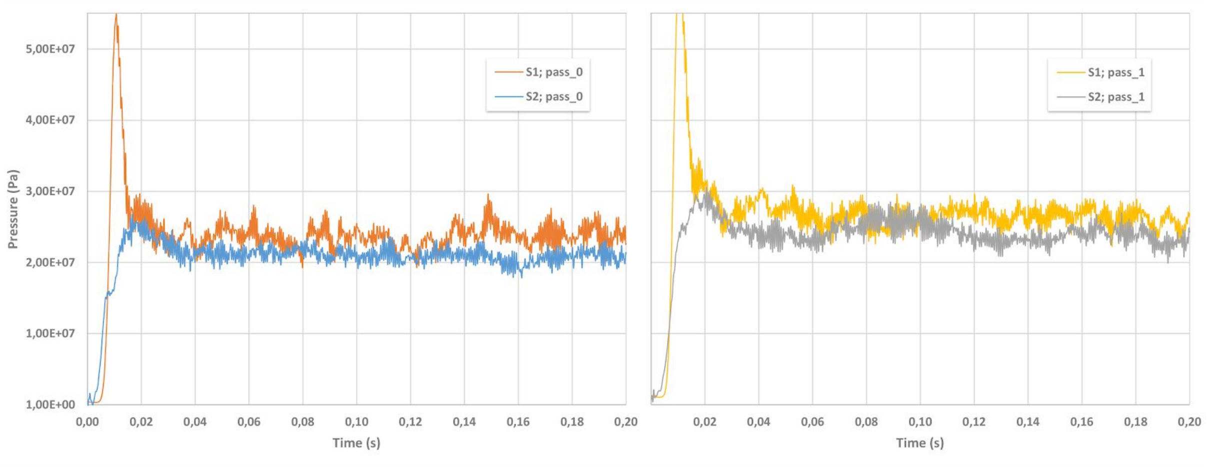

Figure 2: Average pressure at tube inlet. S1: straight tube, S2: helical coil tube, pass_0: initial unexfoliated suspension, pass_1: suspension exfoliated once.

Figure 3 shows the average fluid velocity gradient for S1 and S2, where the fluid velocity is 6.5 % higher for S2 compared to S1.

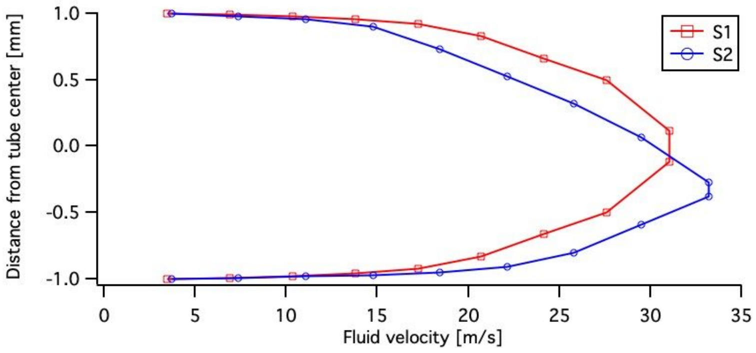

Figure 3: Average fluid velocity gradient for S1 and S2 at pass_0.

Figure 4 shows the fluid velocity vectors in S1 and S2 for the case of pass_0. A distinct difference between S1 and S2 is the swirling flow in S2 which continues throughout the coil.

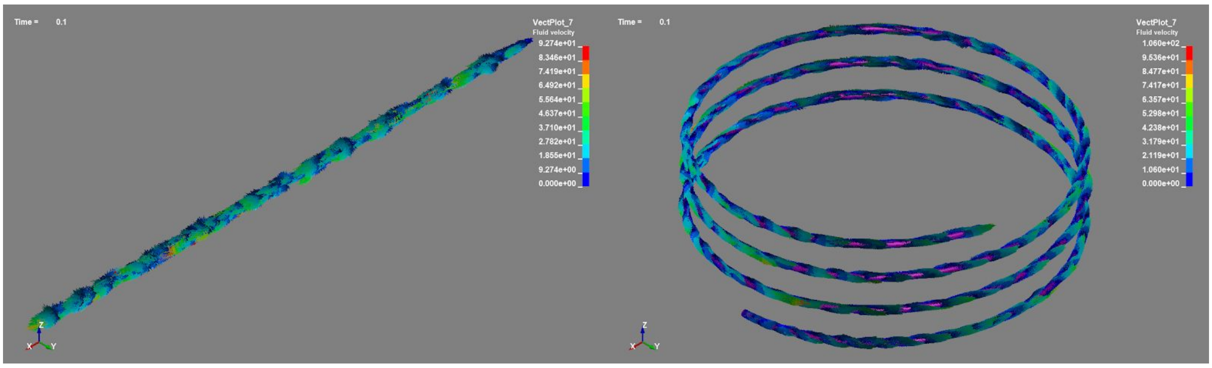

Figure 4: Fluid velocity vectors of S1 (right) and S2 (left) for pass_0.

In Figure 5, the contours of average velocity at the central region of the length of S1 is shown at t = 0.1 s for pass_0. In Figure 6, the contours of average velocity at t = 0.1 of S2 is shown at a region far from the tube entrance for pass_0. Comparison of these two figures reveals details of flow pattern in two geometries: on average, the velocity profile gradient in the straight tube is parabolic with a maximum at the centerline of the tube while the velocity profile in the helical coil tube has a maximum close to the tube wall at the side of high helix radius. This results in a preferred shear surface with high wall shear stress in S2 but not in S1.

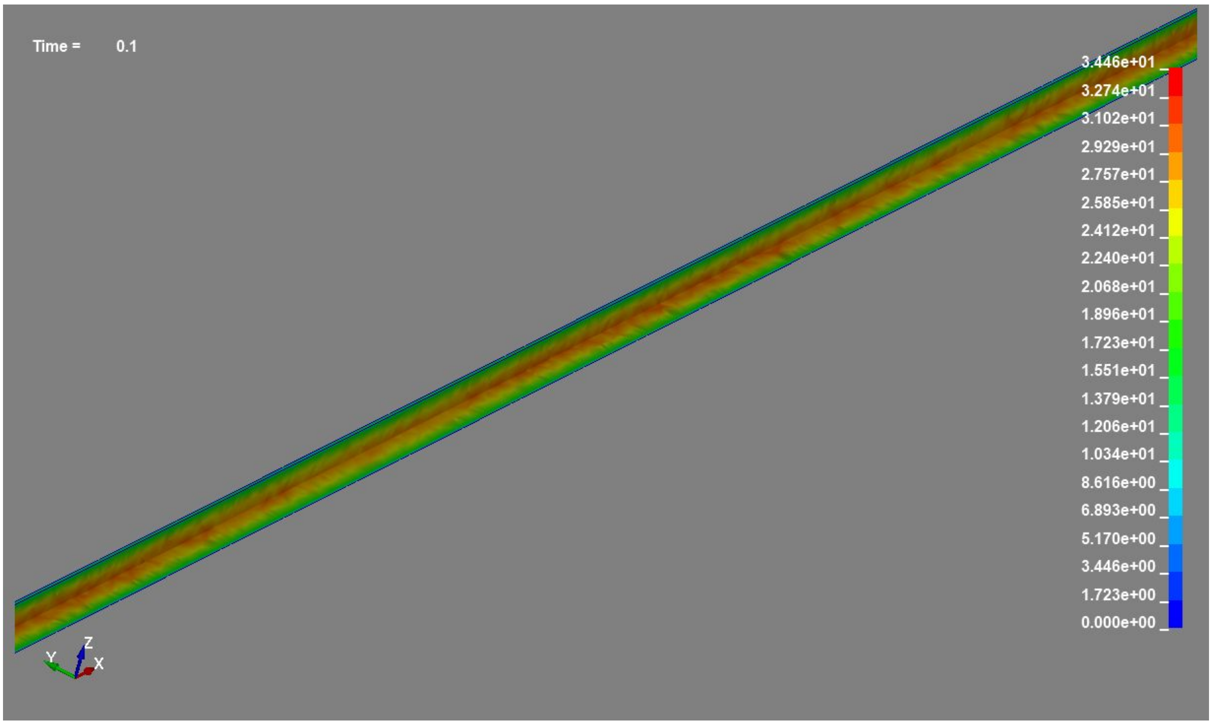

Figure 5: Contours of average velocity on two perpendicular planes at the central region of the straight tube for pass_0. The unit of the scale bar is ms^{-1}.

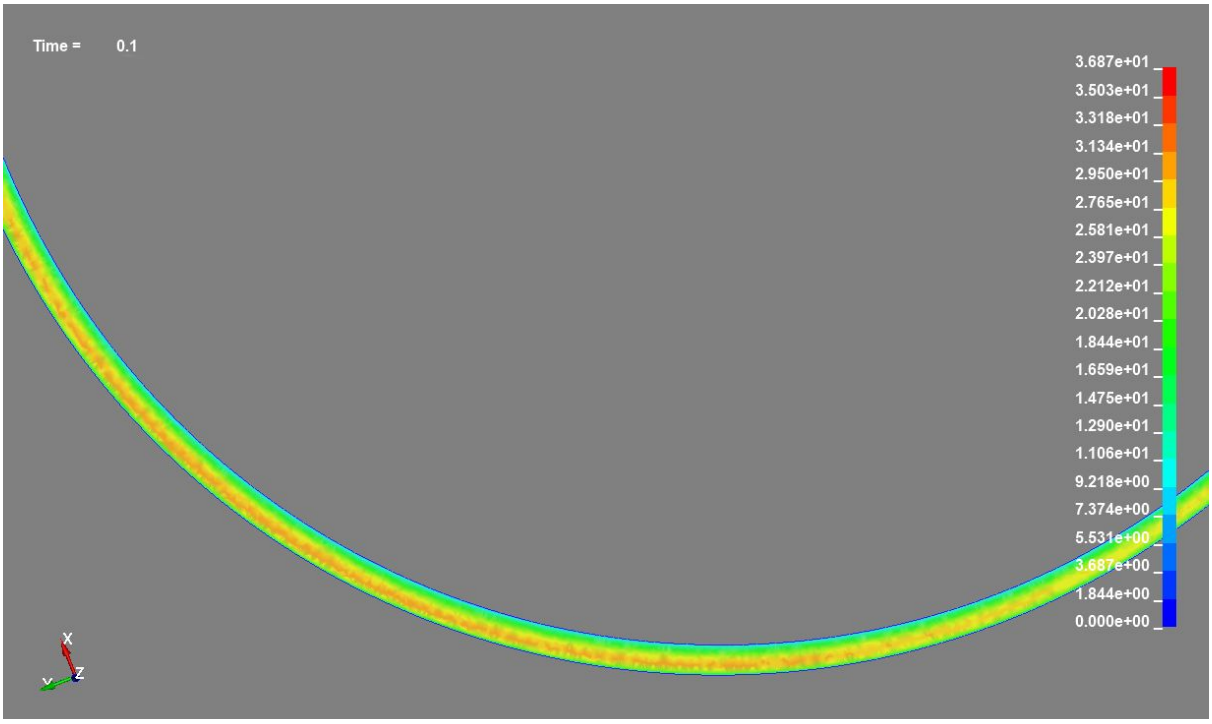

Figure 6: Contours of average velocity on a plane normal to Z-axis through the helical coil tube at region far from the entrance for pass_0. The unit of the scale bar is ms^{-1}.

Figure 7 and 8 show the contours of average shear stress vectors on the surface of S1 and S2 at two times t = 0.1 and t = 0.15 for pass_0. In S1 the distribution of shear vectors do not show any order, however, the pattern remains approximately the same through the time. The distribution of shear vectors in S2, however, reveals a distinct difference between the surface adjacent to the lowest helix radius (r_i) and the surface adjacent to the highest helix radius (r_o), i.e, high shear stress toward r_o and low shear stress toward r_i. In S2 the average surface shear stress pattern is constant through the time. In both S1 and S2, an initial, short length at the entrance side are exempt from the general behavior.
Simulation results show that the flow behavior did not get affected significantly by the change of viscosity. Accordingly, the results regarding pass_1 are not discussed.

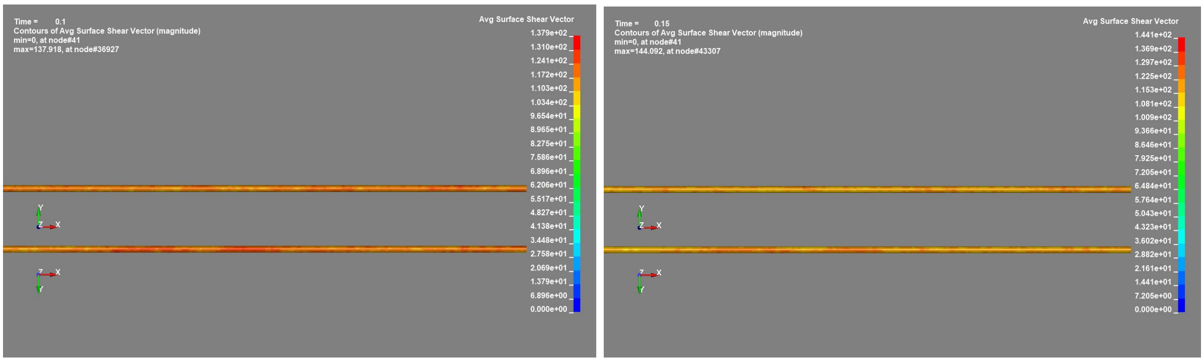

Figure 7: Contours of average shear stress vectors on the surface of S1 for pass_0. The unit of the scale bar is Nm^{-2}.

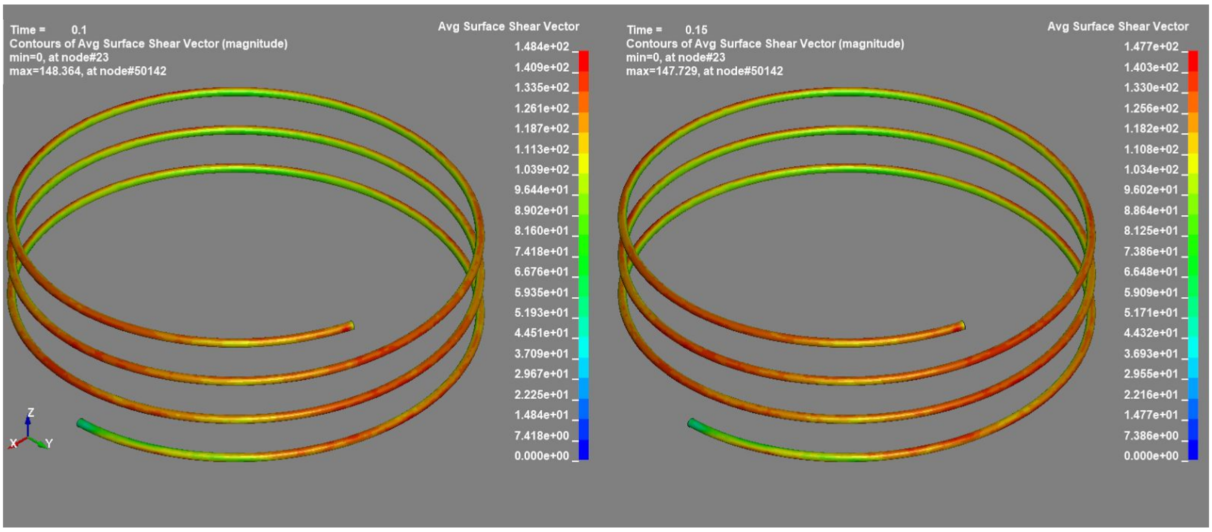

Figure 8: Contours of average shear stress vectors on the surface of S2 for pass_0 (the unit). The unit of the scale bar is Nm^{-2}.

2 Raman spectroscopy

2.1 Flake thickness determination from Raman

Figure 9 shows the ratio I(Si_G) / I(Si_0) as a function of flake thickness. The red + markers shows experimental ratio data from Xiao-Li Li et. al [1] as a function on layer number, N, together with a fitted trend line. The blue ● markers shows experimental data from tape exfoliated HOPG flakes in our Horiba Xplora plus raman system together with the fitted trend line $y = 0,033767 + 0,94954 \cdot e^{-0,072699 \cdot x}$.

When plotting the HOPG particle Raman intensity ratio as a function of thickness instead of layer number we obtain an almost identical trend line as the read-out data from Xiao-Li Li et. al. The offset is approximately a factor 3 which fits well with the graphene layer spacing of 0.333 nm. This offset can be due to the use of a different Raman spectroscopy system compared to Xiao-Li Li et. al. and that no instrumental offset has been subtracted in the AFM measurements in this experiment, which gives a higher thickness value. The trend line from HOPG particle Raman intensity ratio was used to determine the flake thickness distribution of the tube exfoliated flakes from both shear zones.

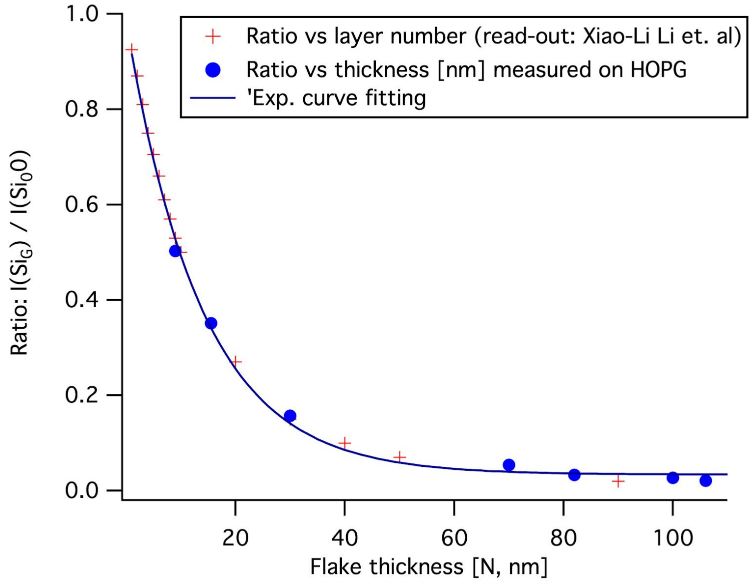

Figure 9: The ratio I(SiG) / I(Si0) as a function of flake thickness. The red (+) markers shows experimental ratio data from Xiao-Li Li et. al [1] as a function on layer number, N, together with a fitted trend line. The blue ($\circ$) markers shows experimental data from tape exfoliated HOPG flakes in the Horiba Xplora plus Raman system as a function of measured thickness in nm together with a fitted trend line.

2.2 Summary of raman spectrums

The raman intensities for each measured particle is summarized in table 1 for S1 and table 2 for S2. all raman spectrums together with optical images of the particles are shown in section 2.3.

Table 1: Raman intensity summary for S1.

Image	Position	I_{Si} Flake [counts]	I_{Si} Sub. [counts]	Ratio	Flake size [µm²]	Thickness [nm]
S1-1	1	14433	20617	0,700	3,1	4,9
S1-1	2	16300	20206	0,807	1,1	2,8
S1-1	3	6697	20333	0,329	7,3	16,1
S1-1	4	15158	20636	0,735	5,1	4,2
S1-1	5	13504	19486	0,693	4,3	5,0
S1-2	1	8092	11820	0,685	2,1	5,2
S1-2	2	9534	11718	0,814	2,7	2,7
S1-2	3	1226	11704	0,105	4,1	35,7
S1-2	4	6858	11490	0,597	6,8	7,2
S1-2	5	9925	11543	0,860	2,8	1,9
S1-3	1	4756	15249	0,312	7,4	16,9
S1-3	2	2212	13704	0,161	5,2	27,6
S1-3	3	4856	10808	0,449	1,6	11,4
S1-4	1	7716	12367	0,624	3,5	6,5
S1-4	2	1469	13925	0,105	6,9	35,5
S1-4	3	6305	12991	0,485	1,6	10,2
S1-4	4	9294	13246	0,702	1,9	4,8
S1-4	5	5301	13845	0,383	6,2	13,8
S1-5	1	3096	11018	0,281	28,4	18,5
S1-5	2	1724	11045	0,156	38,2	28,2
S1-6	1	3570	5549	0,643	3,0	6,1
S1-6	2	2559	5523	0,463	1,5	10,9
S1-6	3	4045	5216	0,775	3,4	3,4
S1-6	4	3013	5031	0,599	1,4	7,1
S1-6	5	453	5059	0,090	18,5	39,0
S1-7	1	11267	15281	0,737	9,2	4,1
S1-7	2	1978	15311	0,129	25,1	31,6
S1-8	1	1833	5556	0,330	2,0	16,0
S1-8	2	739	5243	0,141	5,7	30,0
S1-8	3	380	5340	0,071	14,9	44,5
S1-10	1	638	6495	0,098	2,9	37,0
S1-11	1	1499	10133	0,148	3,8	29,1
S1-12	1	3443	10285	0,335	1,6	15,8
S1-13	1	1898	10169	0,187	4,0	25,1
S1-14	1	709	9561	0,074	10	43,4
S1-15	1	2061	11412	0,181	3,2	25,7
S1-16	1	1328	12947	0,103	6,7	36,1
S1-17	1	2093	10802	0,194	3,2	24,5
S1-18	1	2995	9303	0,322	1,4	16,4
S1-19	1	1041	7837	0,133	6,0	31,1
S1-19	2	463	9606	0,048	18	57,6
S1-19	3	993	9608	0,103	19,1	35,9
S1-19	4	2617	9608	0,272	2,2	19,0
S1-19	5	541	10091	0,054	9,7	53,2
S1-19	6	941	9667	0,097	306	37,2

Table 2: Raman intensity summary for S2.

Image	Position	I_{Si} Flake [counts]	I_{Si} Sub. [counts]	Ratio	Flake size [µm²]	Thickness [nm]
S2-1	1	1479	10568	0,140	13,2	30,1
S2-1	2	731	10503	0,070	8,4	45,1
S2-1	3	4450	10237	0,435	2,8	11,9
S2-1	4	9142	10581	0,864	2,0	1,8
S2-2	1	10333	11283	0,916	1,2	1,0
S2-3	1	655	13300	0,049	497	56,6
S2-4	1	1325	12094	0,110	9,8	34,8
S2-4	2	640	11753	0,054	7,4	52,6
S2-4	3	9011	11721	0,769	1,6	3,5
S2-5	1	1526	14516	0,105	27,2	35,6
S2-6	1	1939	13734	0,141	3,1	30,0
S2-7	1	6329	12997	0,487	5,9	10,2
S2-8	1	4678	8099	0,578	1,8	7,7
S2-8	2	3078	7877	0,391	2,9	13,5
S2-8	3	1305	7333	0,178	3,2	25,9
S2-8	4	243	7176	0,034	31,6	126,6
S2-9	1	296	5668	0,052	36,8	54,2
S2-9	2	1111	5544	0,200	5,2	23,9
S2-9	3	575	5502	0,105	10,1	35,7
S2-9	4	1184	5199	0,228	14,0	21,8
S2-9	5	544	5178	0,105	7,2	35,6
S2-9	6	667	5425	0,123	5,4	32,5
S2-9	7	681	5438	0,125	5,1	32,2
S2-9	8	509	5475	0,093	7,2	38,2
S2-10	1	2748	8359	0,329	12,0	16,1
S2-10	2	898	8330	0,108	36,0	35,1
S2-11	1	1382	14067	0,098	8,2	37,0
S2-12	1	2674	9161	0,292	5,3	17,9
S2-13	1	7786	9757	0,798	1,5	3,0
S2-14	1	1037	13638	0,076	98,4	42,8
S2-15	1	863	10183	0,085	43,7	40,2
S2-16	1	624	10798	0,058	391	50,6
S2-17	1	3392	10848	0,313	1,9	16,9
S2-19	1	4446	10345	0,430	2,0	12,0
S2-20	1	9099	11237	0,810	1,7	2,8
S2-21	1	2842	10053	0,283	3,3	18,4
S2-22	1	6169	10219	0,604	5,0	7,0
S2-23	1	1070	10291	0,104	11,2	35,8
S2-24	1	1904	10949	0,174	58,0	26,3
S2-25	1	632	10992	0,057	165	50,7
S2-25	2	986	10988	0,090	66,3	38,9
S2-25	3	480	10923	0,044	32,3	62,4
S2-25	4	753	10724	0,070	44,0	44,8
S2-25	5	1961	10861	0,181	10,5	25,7
S2-26	1	1055	9865	0,107	272	35,3

S1-19	7	548	9667	0,057	15,7	51,2
S1-19	8	659	9667	0,068	24,7	45,6
S1-20	1	1030	10113	0,102	12,4	36,2
S1-20	2	737	9948	0,074	91,8	43,5
S1-20	3	746	10061	0,074	61,6	43,4
S1-20	4	345	10209	0,034	278	144,1
S1-20	5	336	9929	0,034	29,7	130,3

S2-27	1	1177	9503	0,124	78,0	32,4
S2-28	1	5060	9849	0,514	1,6	9,4
S2-28	2	1357	9849	0,138	9,8	30,4
S2-29	1	923	10951	0,084	53,0	40,4
S2-30	1	1513	10484	0,144	61,0	29,6
S2-31	1	4788	9643	0,497	1,03	9,9
S2-31	2	4926	9580	0,514	6,94	9,4
S2-31	3	1155	9240	0,125	4,32	32,2
S2-31	4	2071	9421	0,220	2,71	22,4

Some flakes were also measured in AFM to check the accuracy of the method. Flake S2-9 1 were measured to be 54.2 nm in Raman and by AFM 55 nm in centre and 27 nm at edges. Flake S2-9 3 were measured to be 35.7 nm in Raman and by AFM 40 nm in centre and 18 nm at edges. Flake S2-9 4 were measured to be 21.8 nm in Raman and by AFM 25 nm in centre and 20 nm at edges. Since the raman value is a mean value of three points across each flake the accuracy of this method can be considered high.

2.2.1 Raman spectra for S1 and S2

This section contains raman spectrums for flakes exfoliated by both S1 and S2. Each diagram shows $I(Si_G)$ as a solid line and $I(Si_0)$ as a dotted line. The name of each spectra corresponds to the name of the optical image and flake number found in section 2.2.2. The peak between 900 and 100 cm-1 is a reflection in the system and belongs to the background. This peak shrinks with thicker flakes.

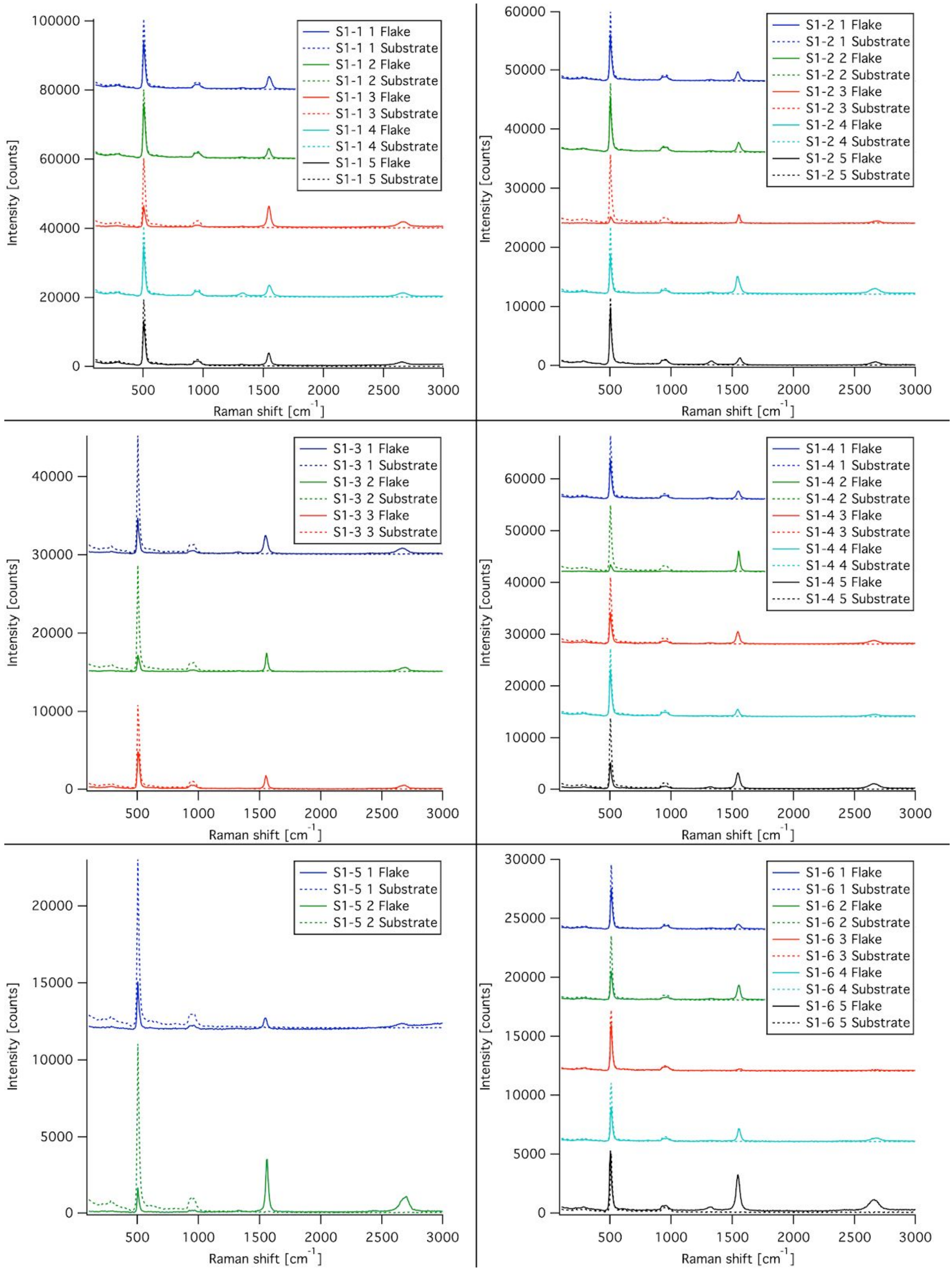

Figure 10: Raman spectrums from exfoliated flakes in image S1-1 to S1-6.

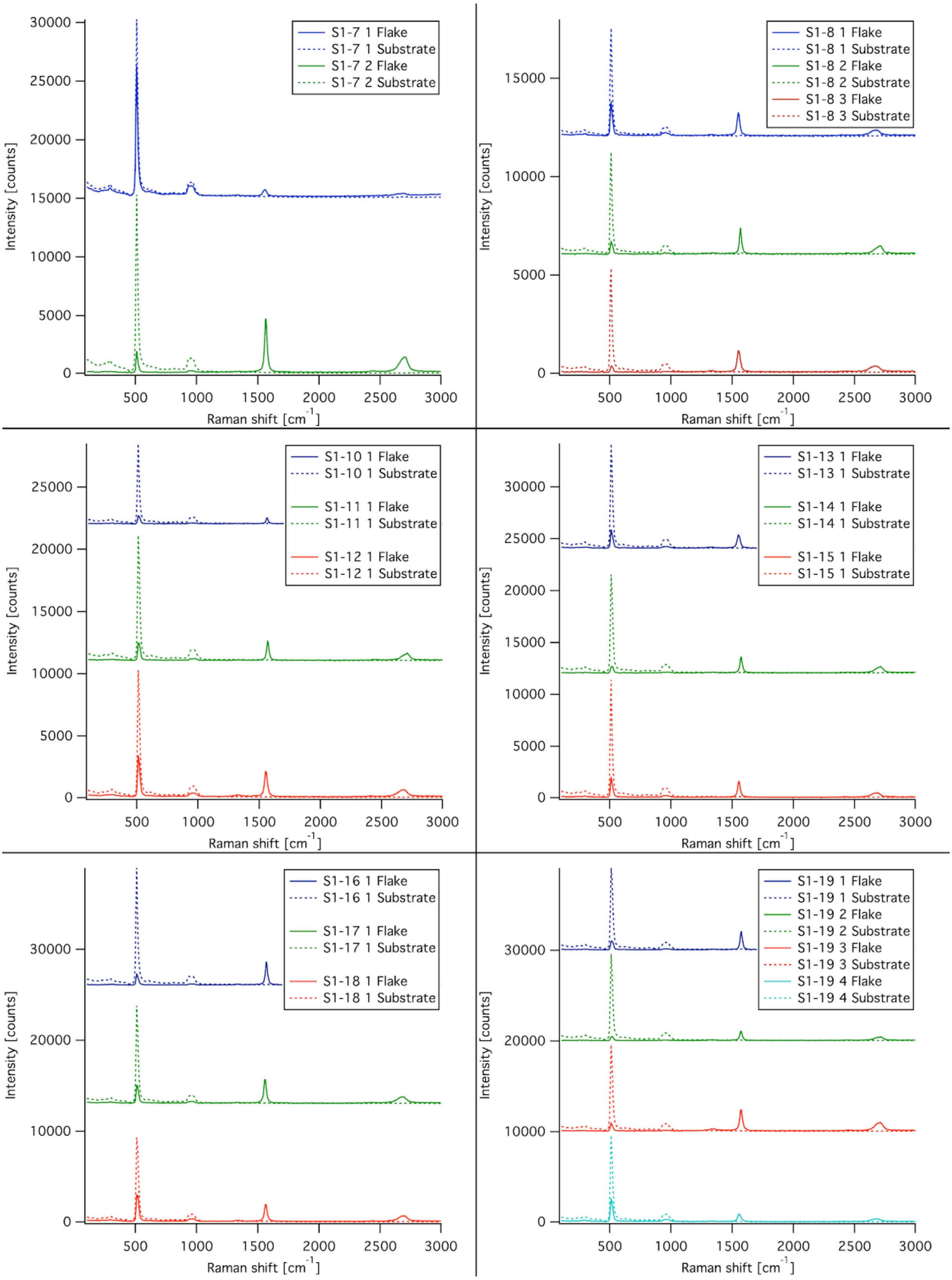

Figure 11: Raman spectrums from exfoliated flakes in image S1-7 to S1-19.

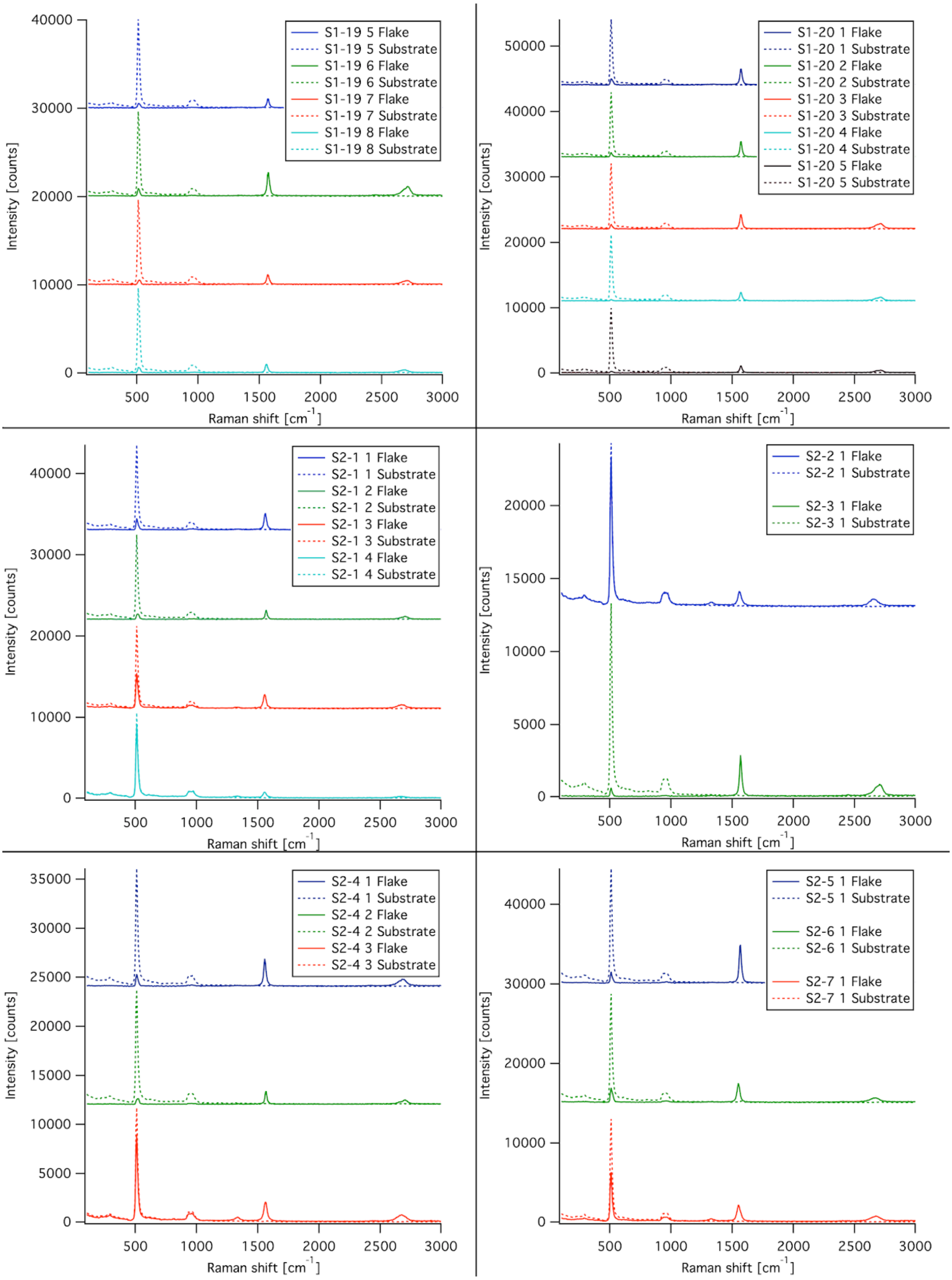

Figure 12: Raman spectrums from exfoliated flakes in image S1-19 to S2-7.

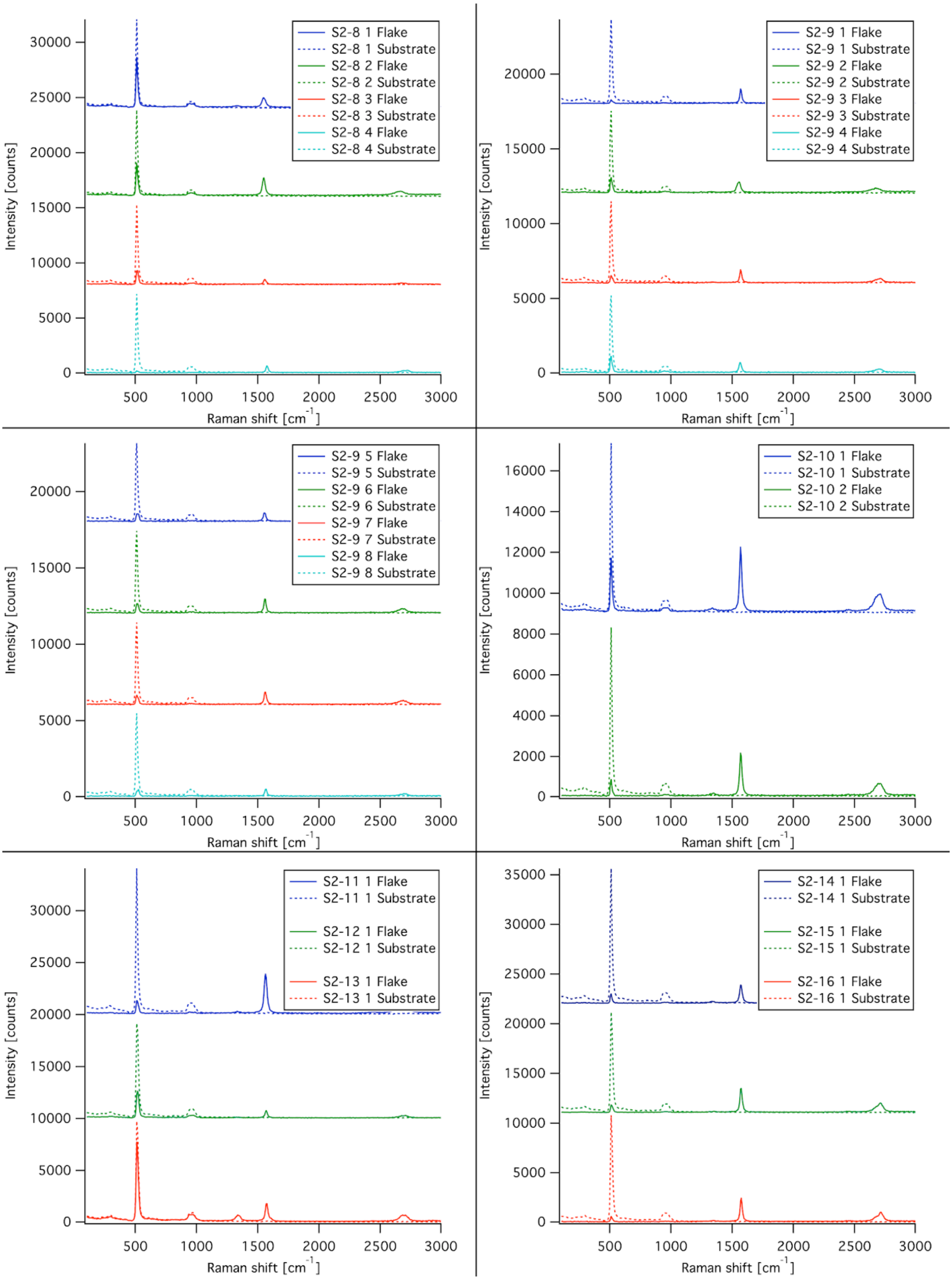

Figure 13: Raman spectrums from exfoliated flakes in image S2-8 to S2-16.

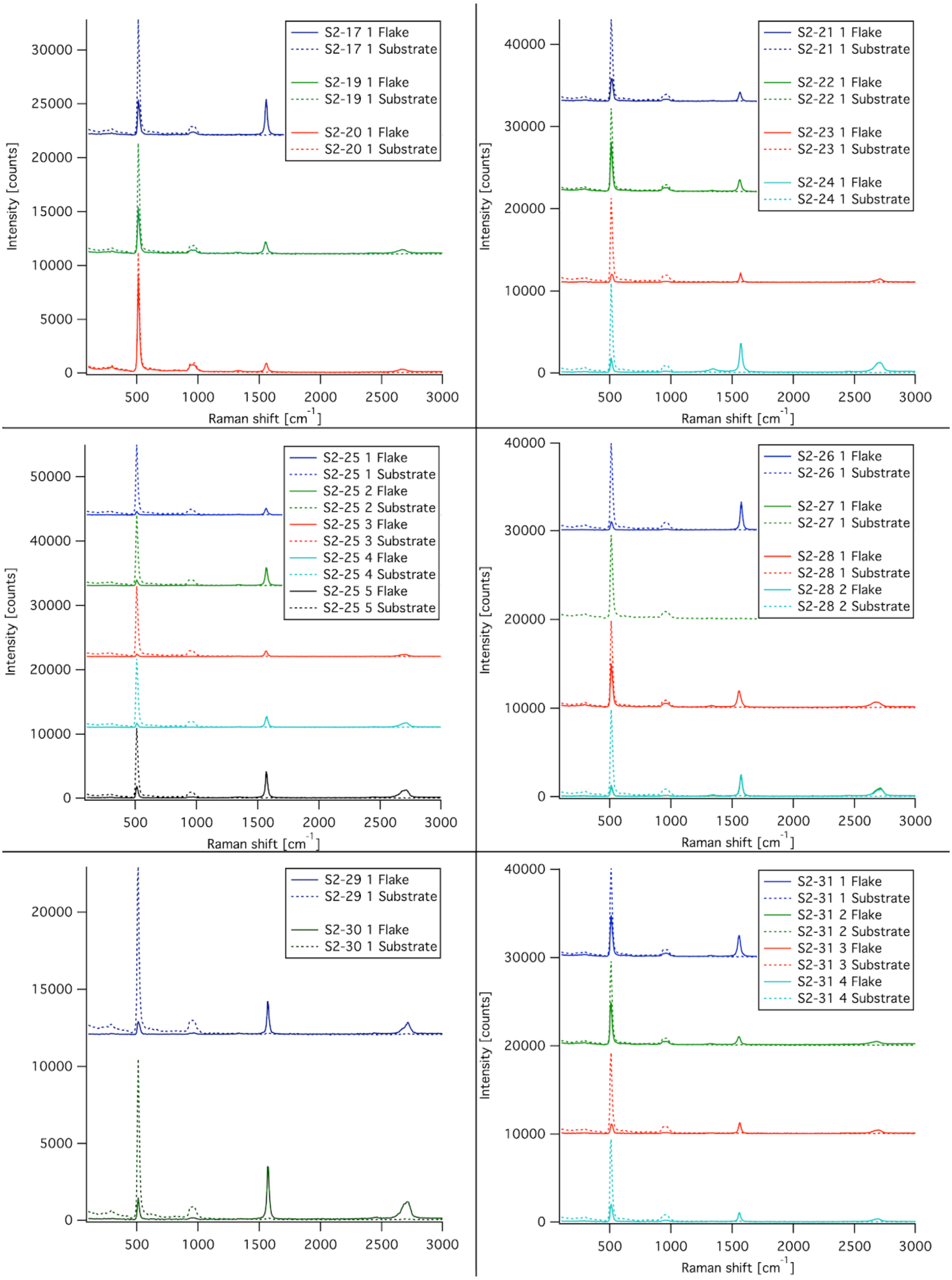

Figure 14: Raman spectrums from exfoliated flakes in image S2-20 to S2-31.

2.2.2 Optical images of the measured particles in raman

This section contains optical images, achieved from the raman microscope, for flakes exfoliated by both S1 and S2. Each image and flake are numbered and the corresponding spectra is found in section 2.2.1. The magnification is x1000 in all images.

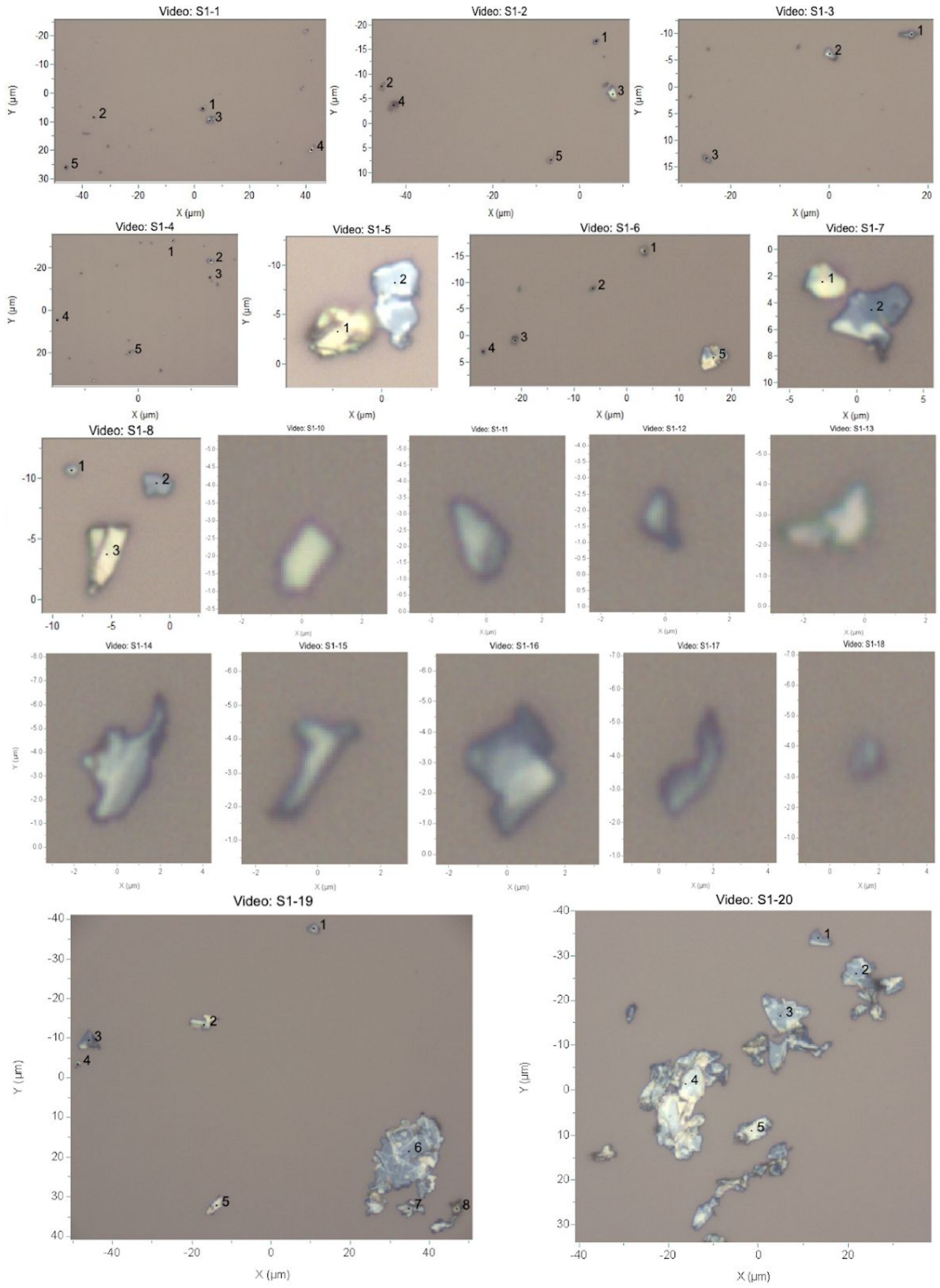

Figure 15: Optical images from Raman microscope with 1000x magnification showing images S1-1 to S1-20.

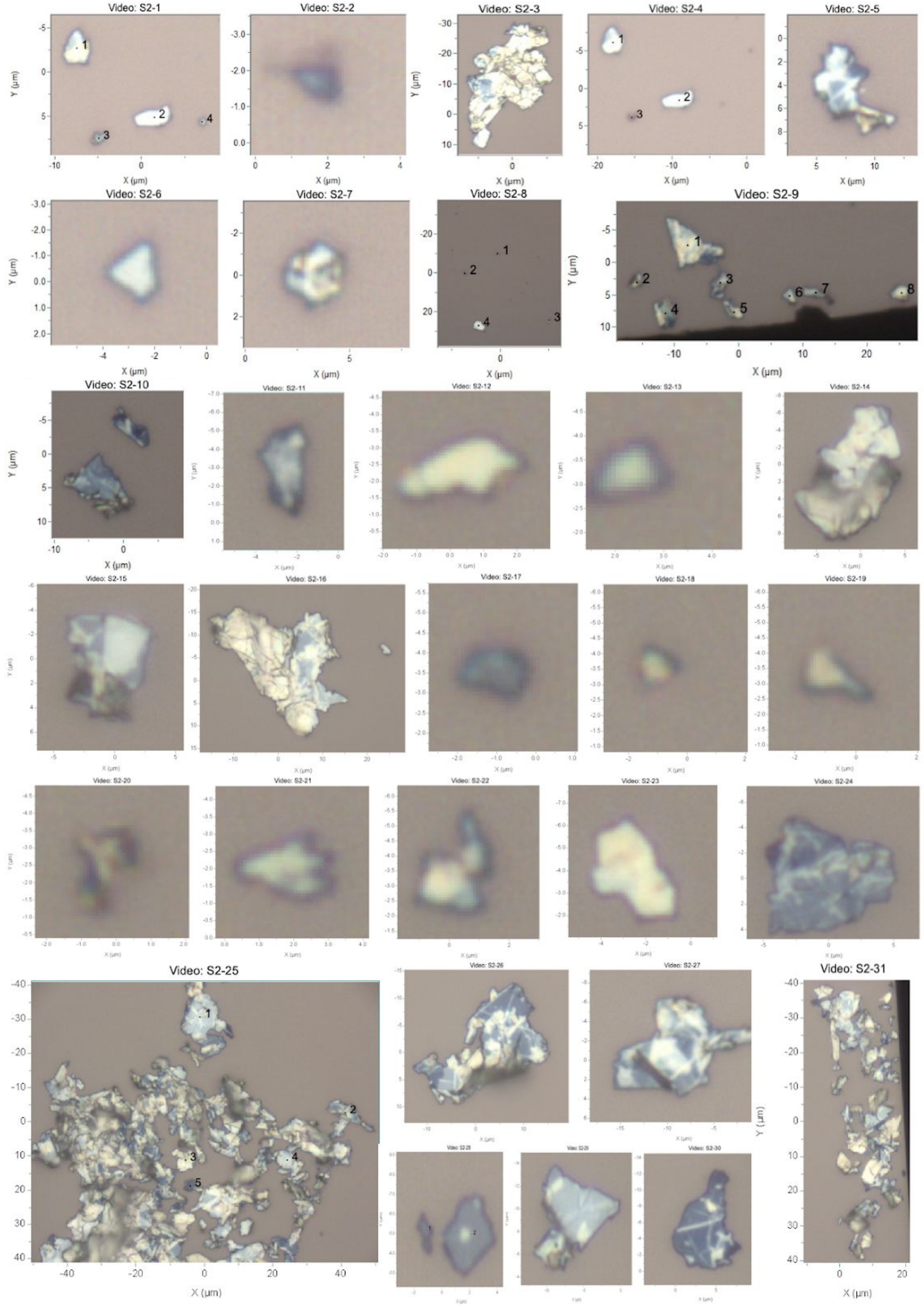

Figure 16: Optical images from Raman microscope with 1000x magnification showing images S2-1 to S2-31.

3 SEM characterization

3.1 SEM imaging

The flake size distribution was investigated using a field emission scanning electron microscope (TESCAN MAIA3-2016) at 3 kV. The 1 cm^2 silicon wafer were divided into 9 (3x3 mm) sections (3 rows and 3 columns). To obtain a fair representation of the flakes the center and left center section was imaged. Each section was analyzed with 900 (30x30) images using a view field of 100 μm (magnification: 5537x). The SEM instrumental resolution was 1 nm and the exfoliated material had a flake length and width in the range from approximately 100 nm to 10 μm. The SEM viewfield and image resolution was set to generate a pixel size of 49 nm, which was sufficient to determine the flake size distribution by image analysis. A total amount of 2648 flakes was analyzed. Figure 17 to Figure 19 shows a small selection of the SEM images used for determination of the flake size distribution.

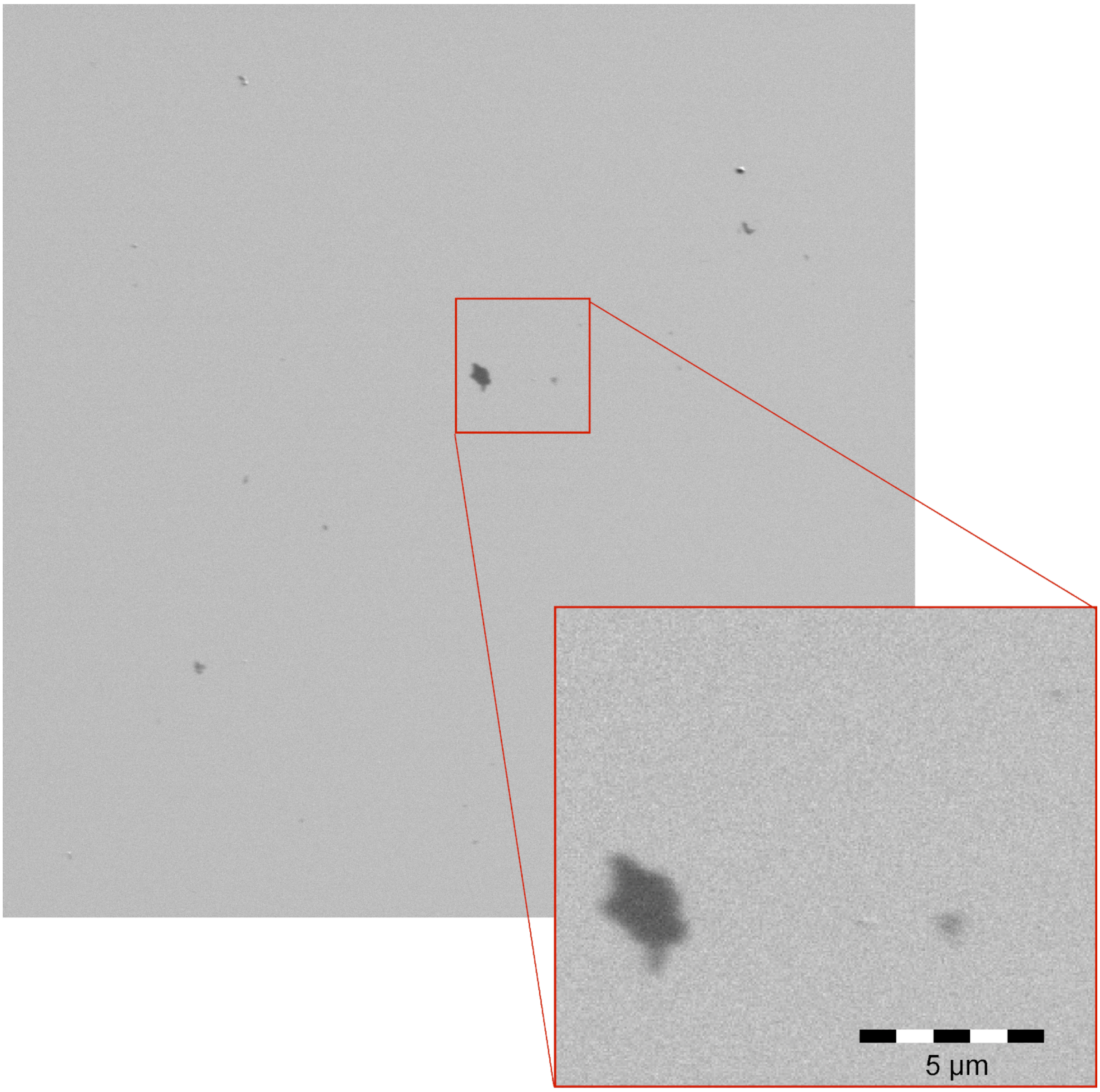

Figure 17: SEM image of flakes exfoliated in S1, the viewfield is 100 μm (magnification: 5537x). The viewfield of the cut-out is 15 μm and the scale bar is 5 μm.

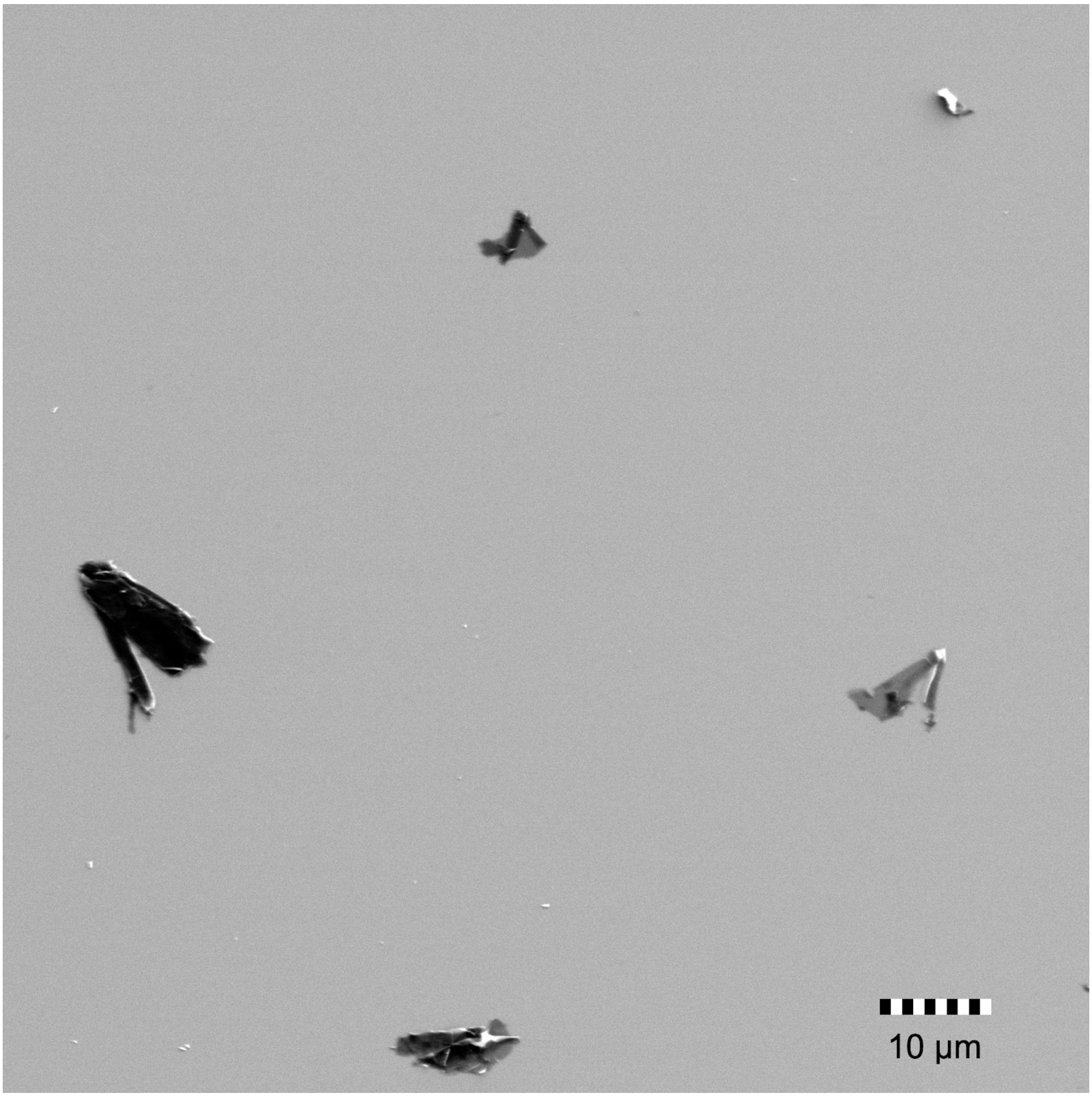

Figure 18: SEM image of large flakes exfoliated in S1, the viewfield is 100 µm (magnification: 5537x).

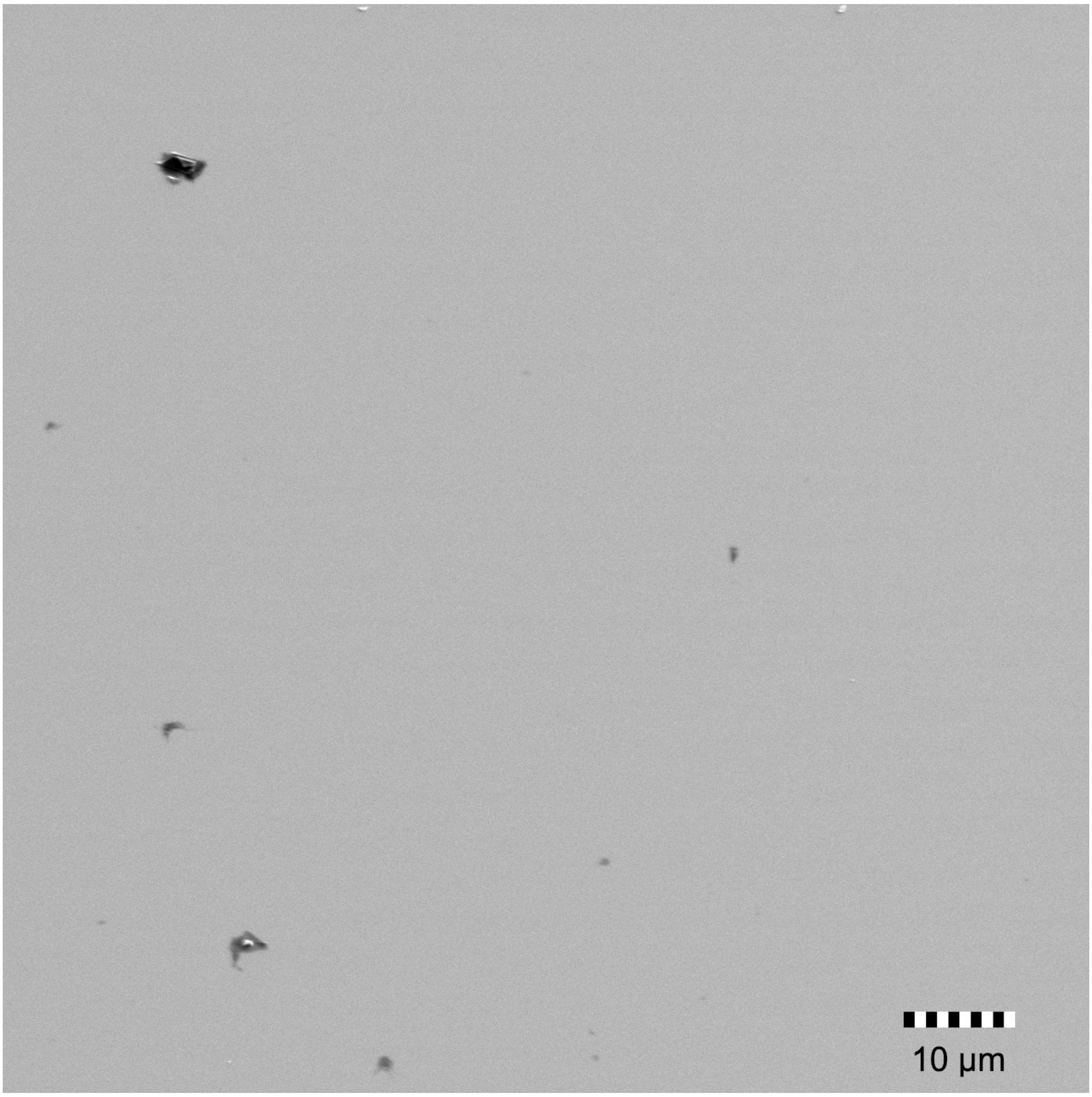

Figure 19: SEM image of flakes exfoliated in S1, the viewfield is 100 µm (magnification: 5537x).

3.2 Image analysis

In order to determine the flake size distribution the particles were characterized in size by image analysis using ImageJ software. Contrast and brightness settings during image acquisition of all SEM-images were held constant to simplify the grayscale thresholding. Before thresholding, the images were filtered three times using a Median filter with size 1 pixel. Most flakes were simple to separate from the background due to the substantial difference in intensity values, and the automatic thresholding plugin RenyiEntropy was best suited for our 16-bit images. Some of the flakes that were not thin and flat in the SEM-images had both dark and white area and therefore the grayscale thresholding was insufficient. These flakes were removed from the automated imageJ algorithm and processed manually. For those flakes we used an edge detection procedure instead of grayscale thresholding. Figure 20 shows the difference between the two methods.

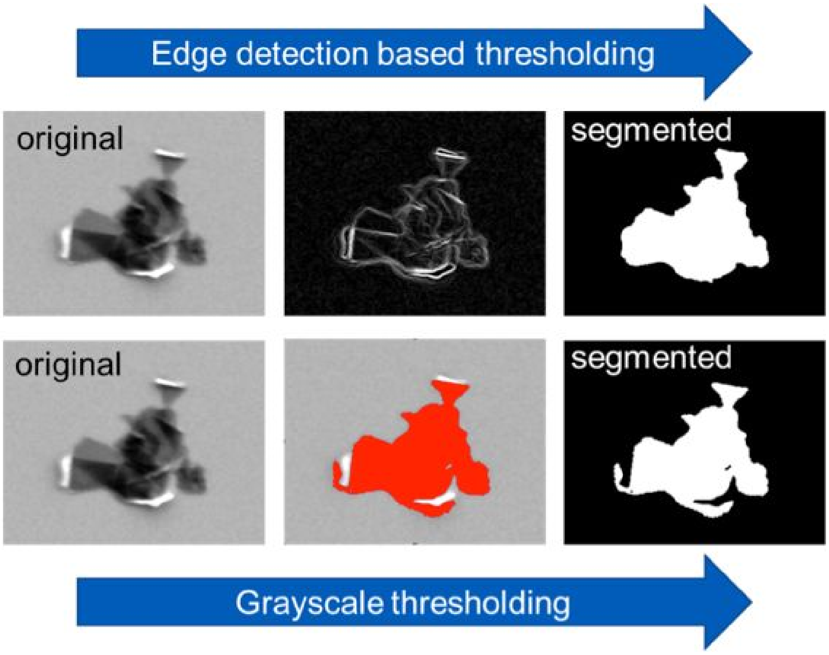

Figure 20: Difference between edge detection thresholding and grayscale thresholding.

In ImageJ the function “Find Edges” were used followed by automatic thresholding to obtain a binary image. After image inversion, the edges needed to be dilated to “close” eventual openings, followed by “fill holes” and finally eroding. Since the edge detected was 1 pixel offset, i.e. gave an increased radius with 1 pixel, we used the eroding one additional time to avoid overestimation of the area.

3.2 Initial material

The initial material used was thermally expanded graphite from Graphit Kropfmühl in Germany (EXG 9840). To thermally expand graphite the layered structure of graphite is intercalated by sulphur or nitrogen salts followed by rapid heating. Under the influence of heat the layers separate like an accordion, and the graphite flakes expand. Depending on degree of intercalation due to intercalation agent, time heating temperature etc. the expanded graphite gets different fractions of grains in various sizes and crystallinity.

From SEM backscatter images, figure 21, it can be seen that the large EXG 9840 particles contains grains with a size is in the range of 10 to 100 square micrometer and thickness from hundred to a few hundred nanometers.

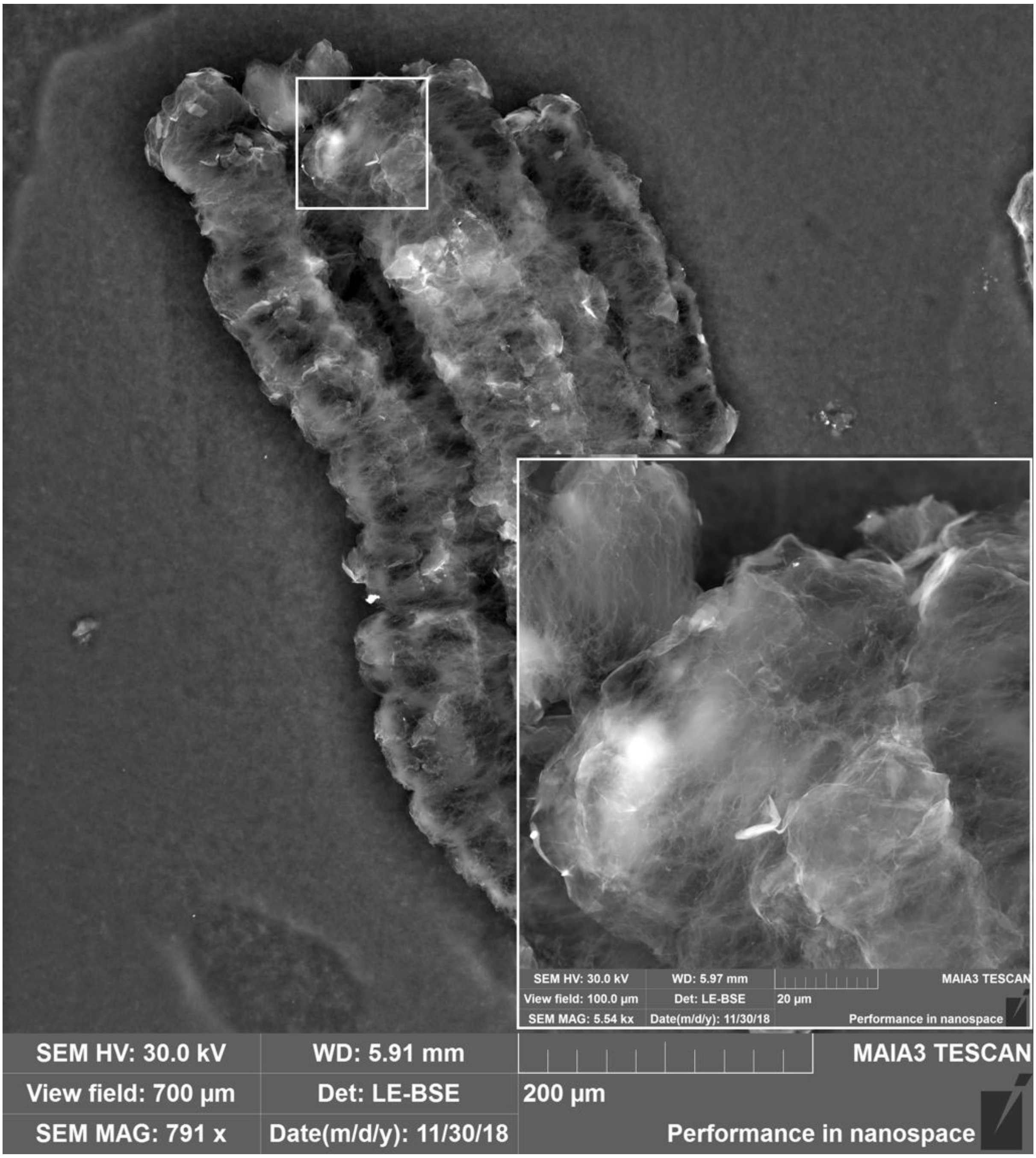

Figure 21: SEM backscatter image of the thermally expanded graphite.

References

1. Li, X.-L. et al. Layer number identification of intrinsic and defective multilayered graphenes up to 100 layers by the raman mode intensity from substrates. Nanoscale **7**, 8135–8141 (2015).
